# The desmosomal cadherin desmoglein-3 acts as a keratinocyte anti-stress protein via suppression of p53

**DOI:** 10.1038/s41419-019-1988-0

**Published:** 2019-10-03

**Authors:** Ambreen Rehman, Yang Cai, Christian Hünefeld, Hana Jedličková, Yunying Huang, Muy Teck Teh, Usama Sharif Ahmad, Jutamas Uttagomol, Ying Wang, Angray Kang, Gary Warnes, Catherine Harwood, Daniele Bergamaschi, Eric Kenneth Parkinson, Martin Röcken, Hong Wan

**Affiliations:** 1 Centre for Oral Immunobiology and Regenerative Medicine, Institute of Dentistry, Barts and The London, School of Medicine and Dentistry, Queen Mary University of London, London, UK; 2 CB Joint MHNCRL, Hospital and School of Stomatology, Guizhou Medical University, Guiyang, China; 3 Department of Dermatology, Eberhard Karls University, Tübingen, Germany; 4 First Department of Dermatovenerology, St. Anne’s Faculty Hospital, Brno, Czech Republic; 5 Centre for Cell Biology and Cutaneous Research, Blizard Institute, Barts and The London, School of Medicine and Dentistry, Queen Mary University of London, London, UK; 6 Present Address: Department of Dermatology, Charité - Universitätsmedizin Berlin, 10117 Berlin, Germany

**Keywords:** Stress signalling, Molecular biology

## Abstract

Desmoglein-3 (Dsg3), the *Pemphigus Vulgaris (PV)* antigen (PVA), plays an essential role in keratinocyte cell–cell adhesion and regulates various signaling pathways involved in the progression and metastasis of cancer where it is upregulated. We show here that expression of Dsg3 impacts on the expression and function of p53, a key transcription factor governing the responses to cellular stress. Dsg3 depletion increased p53 expression and activity, an effect enhanced by treating cells with UVB, mechanical stress and genotoxic drugs, whilst increased Dsg3 expression resulted in the opposite effects. Such a pathway in the negative regulation of p53 by Dsg3 was Dsg3 specific since neither E-cadherin nor desmoplakin knockdown caused similar effects. Analysis of Dsg3^−/−^ mouse skin also indicated an increase of p53/p21^WAF1/CIP1^ and cleaved caspase-3 relative to Dsg3^+/−^ controls. Finally, we evaluated whether this pathway was operational in the autoimmune disease PV in which Dsg3 serves as a major antigen involved in blistering pathogenesis. We uncovered increased p53 with diffuse cytoplasmic and/or nuclear staining in the oral mucosa of patients, including cells surrounding blisters and the pre-lesional regions. This finding was verified by in vitro studies where treatment of keratinocytes with PV sera, as well as a characterized pathogenic antibody specifically targeting Dsg3, evoked pronounced p53 expression and activity accompanied by disruption of cell–cell adhesion. Collectively, our findings suggest a novel role for Dsg3 as an anti-stress protein, via suppression of p53 function, and this pathway is disrupted in PV.

## Introduction

Desmoglein-3 (Dsg3), a cadherin superfamily member, is an adhesion protein in desmosomes. Recent evidence suggests that Dsg3 acts as a regulator of various pathways governing cell adhesion, proliferation, differentiation, morphogenesis, and migration^1–7^. However, the function of non-junctional Dsg3 remains poorly understood. In the skin, Dsg3 is largely restricted to the basal layer of the epidermis, while in oral mucosa uniform expression occurs across stratified squamous epithelia^8,9^. Why these different distribution patterns exist is unknown.

Dsg3 is down-regulated in PV where Dsg3 serves as a major antigen (PVA) for autoantibodies, causing disruption of cell–cell cohesion and pemphigus acantholysis in Dsg3-expressing tissues^10–14^. However, other studies suggest PV is caused by autoantibodies that target non-Dsg receptors, triggering intracellular signaling and consequently cell apoptosis, leading to blistering^14–16^. Despite numerous studies, the pathogenesis of PV remains an issue of debate^15,17^.

Dsg3 is upregulated in cancer with its exact role remains uncertain^1^. In vitro gain-of-function experiments support its pro-cancerous role; overexpression of Dsg3 elicited pronounced membrane protrusions and augmented cell migration via activation of various pathways^1,3–5,18^. Conversely, Dsg3 depletion resulted in inhibition of tumor growth and metastasis^19^.

Our preliminary observation, made in MDCK (Madin–Darby canine kidney) cells, showed that dome formation, marking the initiation of epithelial cell differentiation^20–22^ was suppressed by Dsg3 overexpression. Furthermore, overexpression of Dsg3 resulted in the suppression of p53/p21^WAF1/CIP1^, suggesting that Dsg3 could act as an anti-stress protein through negative regulation of p53^23^. p53 is found to be upregulated in some epidermal pathologies, such as psoriasis^24,25^ and lichen planus (LP)^26–28^, though whether any alteration of p53 in PV currently remains unknown. Here, we investigate the hypothesis that Dsg3 counteracts p53 in keratinocytes and explore its potential contribution to the pathogenesis of PV.

## Results

### Dsg3 depletion induces p53 in keratinocytes

**Fig. 1:**
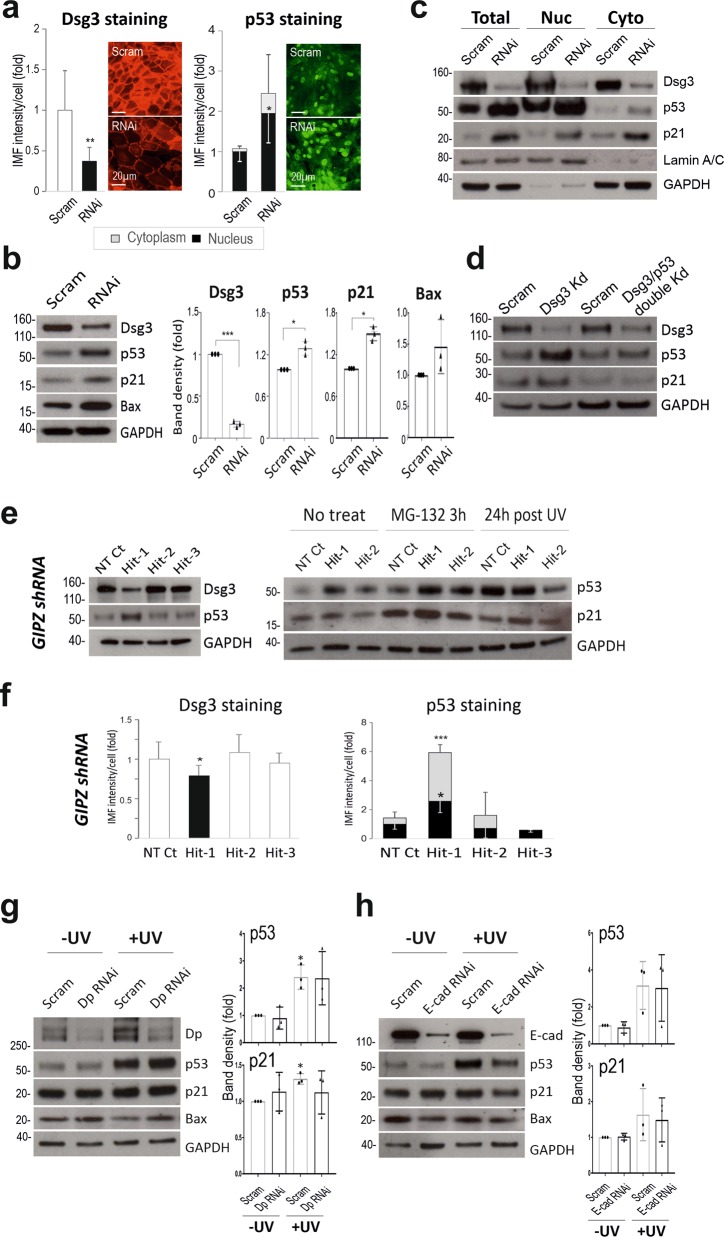
Dsg3 depletion in human keratinocytes enhances p53 expression and activity. **a** Immunofluorescence in NTERTs transiently transfected with Dsg3 specific or scrambled siRNA for 2d showed significantly increased nuclear p53 relative to control (*n* = 7, pooled from 2 independent experiments). Scale bars, 20 µm. **b** Western blotting for p53 and its targets, p21^WAF1/CIP1^/Bax, in NTERTs with Dsg3 knockdown indicated a moderate but significant increase of p53 (*n* = 3–4). **c** Biochemical fractionation of NTERTs with or without Dsg3 knockdown (RNAi). Increased p53 and p21^WAF1/CIP1^ in RNAi samples, especially in the nuclear fraction compared to control. **d** Western blots for the indicated antibodies in lysates with single (Dsg3) and double (Dsg3/p53) knockdown. **e** Western blotting analysis of NTERT cell lines with transduction of GIPZ Lentiviral shRNAs, including non-target (NT) and three hits targeting different regions in the Dsg3 gene. Cells with transduction of one (Hit-1), among three hits, exhibited Dsg3 knockdown with concomitant induction of p53/ p21^WAF1/CIP1^ without and with either MG-132 (25 µM for 3 h) or UVB irradiation, as compared to NT and negative Hit controls. **f** Immunofluorescence analysis indicated a marked increase of p53 in both the nucleus and cytoplasm in cells with transduction of Hit-1 compared to NT and other negative Hit controls. **g**, **h** Western blotting analysis in NTERTs with Dp or E-cadherin knockdown shows distinct protein expression profiles for p53/p21^WAF1/CIP1^/Bax. Cells treated without and with UVB irradiation were shown here (see Dsg3 KD + UV in Fig. 3a below). (mean ± s.d., *p < 0.05, **p < 0.01, ***p < 0.001)

To investigate our hypothesis, we performed an RNAi study in NTERT keratinocytes harboring wild type p53 (wtp53). Knockdown of Dsg3 caused no apparent changes in other junctional proteins including Dsg2 (not shown). Immunofluorescence indicated a significant induction of nuclear p53 in cells with Dsg3 knockdown compared to controls (Fig. 1a). Western blotting detected only a moderate but significant increase of p53 with an increase of its targets p21^WAF1/CIP1^/Bax (Fig. 1b). Lysates of the nuclear and cytoplasmic fractions of the siRNA treated cells were extracted and subjected to Western blotting analysis. Increased p53/p21^WAF1/CIP1^ was evident in the nuclear fraction of RNAi treated cells relative to control (Fig. 1c), confirming augmented p53 levels in cells with Dsg3 depletion. To determine the specificity of Dsg3 RNAi mediated p53 induction, we performed double knockdown for Dsg3/p53 that demonstrated the induction of p53 is mediated by Dsg3 depletion since cells with double knockdown showed attenuation of enhanced p21^WAF1/CIP1^ expression (Fig. 1d). In parallel, we performed Dsg3 knockdown in NTERTs with the lentiviral shRNAs (Dharmacon, USA) targeting three different regions in the Dsg3 gene. We found that one hit rendered Dsg3 knockdown coupled with induction of p53/p21^WAF1/CIP1^ relative to non-target control whereas the other two hits evoked no Dsg3 knockdown and significantly no p53 induction (Fig. 1e). In line with these data, immunofluorescence also detected a significant increase of p53 in both nucleus and cytoplasm in knockdown cells compared to controls (Hit-1 in Fig. 1f). To further evaluate the specificity of the Dsg3-p53 pathway, we performed similar knockdown experiments for desmoplakin, a marker of desmosomes and E-cadherin, a classical cadherin in adherens junctions and found that neither desmoplakin nor E-cadherin depletion evoked comparable effects (Fig. 1g, h, see below).

**Fig. 2:**
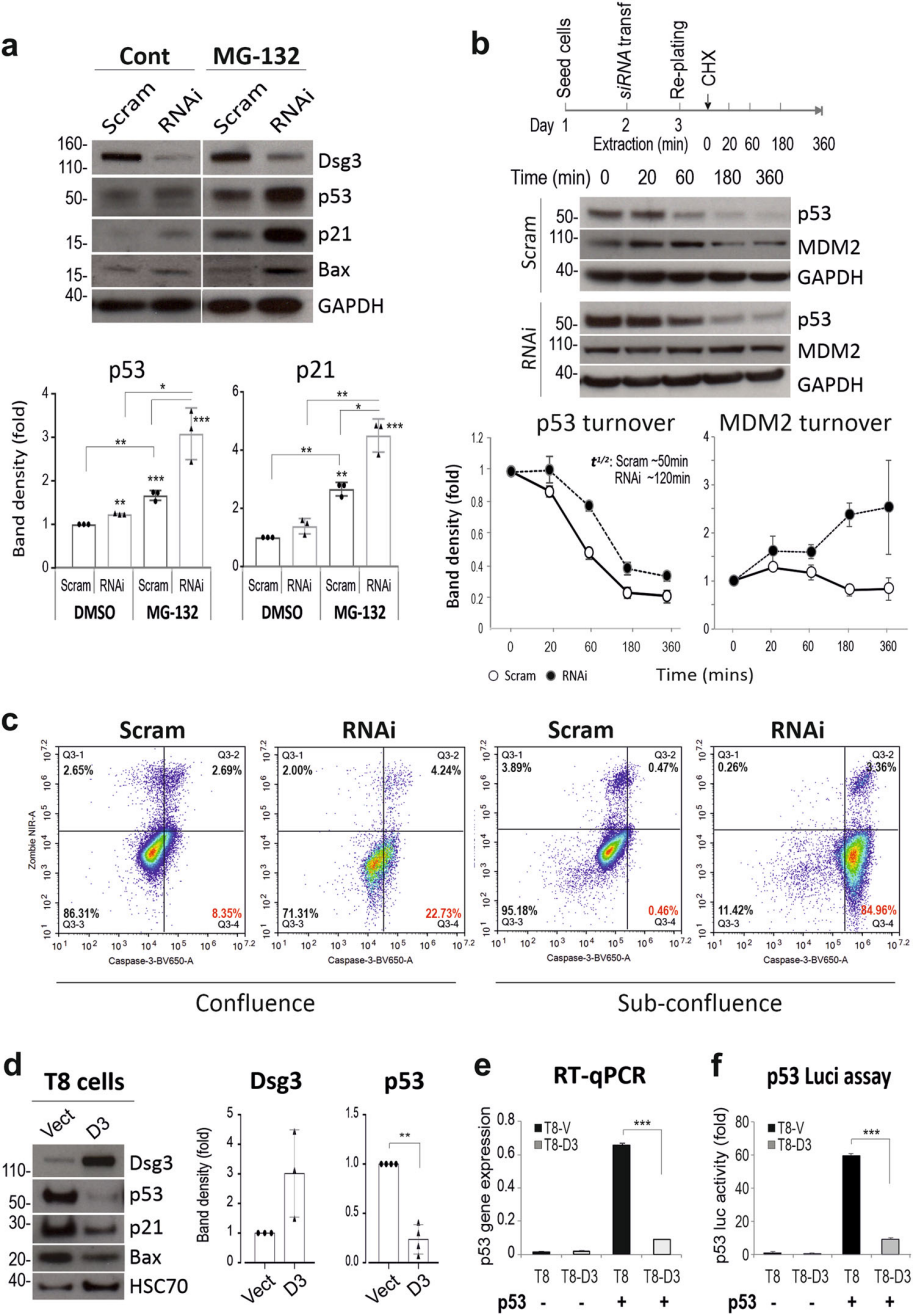
The p53 suppression by Dsg3 was further supported by the inhibition of protein degradation and overexpression of Dsg3. **a** Western blots of siRNA-transfected cells with and without treatment of MG-132 (25 µM) for 3 h. GAPDH and HSC70 were the loading controls. **b** Protein turnover analysis for p53, as well as MDM2, indicated reduced p53 turnover accompanied by MDM2 stabilization in Dsg3 depleted cells. The above is the timeline of the experiment. The band density for each blot was normalized against the loading control in each sample and then against the one at 0 min time point in each condition. The calculated half-life for p53 and MDM2 were shown in the graphs. **c** Flow cytometric analysis of cell viability with live cells (Zombie NIR^−ve^/Caspase-3^−ve^), Zombie NIR^−ve^/Caspase-3^+ve^, both positive (Zombie NIR^+ve^/Caspase-3^+ve^) and Zombie NIR^+ve^/Caspase-3^−ve^ in NTERTs with and without Dsg3 knockdown, grown at 100% or ~40% confluences (the represented data of 3 independent attempts). **d** Protein expression in cutaneous keratinocytes T8 (p53 null, with p53 transfection) Vect control and Dsg3 overexpression (D3) that showed suppression of p53/p21^WAF1/CIP1^ in D3 cells compared to Vect cells. **e** RT-qPCR analysis of p53 expression (mean ± s.e.m.) in T8 cell lines (*n* = 3 independent assays of duplicate in each test). **f** p53 luciferase assay (mean ± s.d.) in T8 cell lines (*n* = 3, a representative of two independent experiments). The comparison was via unpaired two-sided student *t*-test. (**p < 0.01, **p < 0.01, ***p < 0.001)

**Fig. 3:**
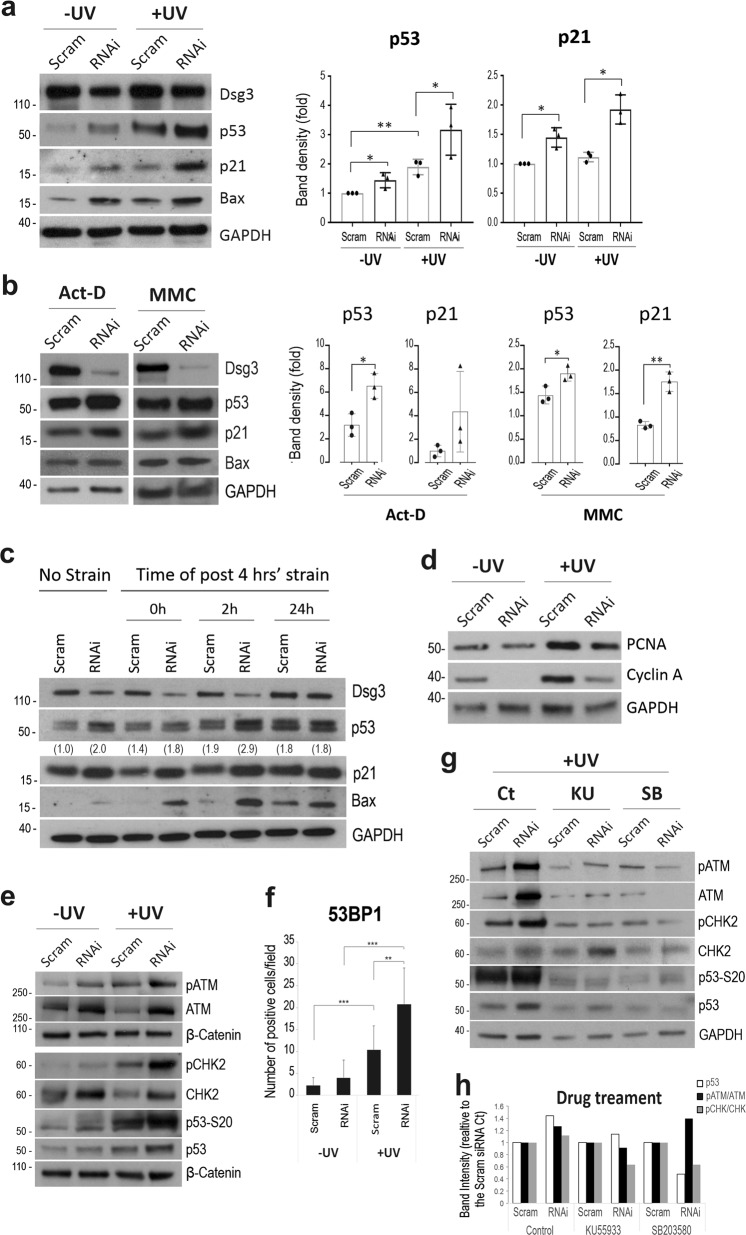
Dsg3 depletion causes further induction of p53 expression and activity in response to stress signals. **a** Western blotting of siRNA pre-treated NTERT cells with and without UVB irradiation for the indicated proteins with the quantitation shown on the right (*n* = 3 biologically independent samples, *p < 0.05). **b** Western blotting for p53/p21^WAF1/CIP1^ in cells treated with or without actinomycin D (Act D, 5 nM) and mitomycin C (MMC, 5 µg/ml) for 24 h, respectively. The quantitation data are shown on the right. Enhanced expression of p53/p21^WAF1/CIP1^ was shown in cells with Dsg3 knockdown and treated with drugs. **c** Mechanical stretching induced increased expression of p53 and p21^WAF1/CIP1^/ Bax in Dsg3 KD cells. The siRNA pre-treated cells were seeded at confluent density in BioFlex plates and then subjected to cyclic strain (TX-5000, 20% amplitude, 1 Hz) for 4 h the following day. Lysates were extracted either immediately after strain or 2 h and 24 h later, respectively, after transferring to a stationary state, along with static control cells. **d** Western blotting analysis for PCNA and cyclin A in siRNA treated cells with and without UV. **e** Western blots for the indicated proteins upstream of p53 as well as phospho-p53-S20 in siRNA pre-treated cells with and without UVB (30 mJ/cm^2^). **f** Qnatitation of 53BP1 nuclear staining (*n* = 10, mean ± S.D., **p < 0.01, ***p < 0.001). **g** Western blotting in siRNA transfected cells treated in the presence and absence of ATM inhibitor KU55933 (20 µM) and p38 MAPK inhibitor SB203580 (20 µM), respectively. All cells were exposed to UVB (30 mJ/cm^2^) in this case. Cells were treated with drugs 1 h before UV and were grown overnight before lysate extraction. **h** The expression of p53 in the same samples as phosphorylated ATM and CHK2 (the last two corrected for total protein) following the indicated drug treatments in Dsg3-depleted cells. The data are expressed as the band intensities in the Dsg3-depleted cells relative to the corresponding scrambled siRNA controls, which were normalized to 1

Because of the highly dynamic nature of p53^29^, we were concerned that moderate changes in p53 in knockdown cells might be masked partially by its rapid turnover. Hence, we treated the siRNA transfected cells with MG-132 (25 µM, 3 h) before protein extraction and a greater increase of both p53 and p21^WAF1/CIP1^ was detected in Dsg3 knockdown cells (Fig. 2a). Similar results were observed in lentivirus shRNA Hit-1 cells compared to controls (Fig. 1e). We also monitored p53 protein turnover in cells treated with cycloheximide (30 µg/ml) by extracting protein at various time points for up to 6 h. As expected, a delayed reduction of p53, accompanied by stabilization of MDM2, a key negative regulator of p53^30^, was found in Dsg3-depleted cells compared to controls (Fig. 2b). p53 half-life calculations indicated the protein existed approximately 2-fold longer in knockdown cells than in control cells (~120 min in RNAi vs ~50 min in control). To test whether the regulation of p53 by Dsg3 had functional consequences we monitored whether there was an increased expression of cleaved/active caspase-3, an established specific marker of epithelial apoptosis^31^. A FACS based Zombie NIR-caspase-3 assay^32^ was performed in siRNA pre-treated cells grown to confluent and sub-confluent conditions that detected a marked increase of positive caspase-3 events (Zombie NIR^−ve^/Caspase-3^+ve^) in Dsg3 knockdown cells as compared to the respective controls; the effects were seen particularly in sub-confluent culture (Fig. 2c). Furthermore, we also detected a reduction of PCNA and Cyclin A that regulate cell cycle progression, in cells with Dsg3 knockdown (see below in Fig. 3d). Collectively, these findings are consistent with our hypothesis that Dsg3 restrains p53 expression and activity.

### Dsg3 overexpression causes suppression of p53 and activity

To confirm our findings, an alternative approach with gain-of-function was conducted in a cutaneous cell line T8 (p53 null) expressing low endogenous Dsg3. T8 cells with transduction of pBABE-hDsg3.myc to overexpress Dsg3 (D3) or pBABE-puro empty vector (Vect), were transiently transfected with a wtp53 plasmid for 2d prior to analysis of p53 expression. We observed marked suppression of p53/p21^WAF1/CIP1^ in D3 line, compared to Vect control, at both the transcript and protein levels (Fig. 2d, e). The p53 transcriptional activity was confirmed by a p53 luciferase assay (Fig. 2f). These results, again, support our hypothesis that Dsg3 negatively regulates p53. To explore the broad role of p53 in cell biology, the influence of Dsg3 modulation (knockdown and ectopic overexpression) on the keratinocyte differentiation program was examined by a series of qPCR analyses for various genes involved in early and late differentiation programs in keratinocytes. To this end, we observed a general inverse relationship between Dsg3 expression and key keratinocyte differentiation markers (Fig. S1). Thus, Dsg3 silencing caused their enhanced expression [premature cell differentiation] whereas the inverse result was detected in Dsg3 overexpressing cells (cell dedifferentiation). These results suggest that the Dsg3-p53 pathway has some influence, at least in part, on the keratinocyte differentiation program.

### Dsg3 constrains p53 in response to stress

Dsg3 expressing tissues, e.g. skin and oral mucosa, are exposed daily to various stresses that could induce p53^23^. Thus, we challenged cells with various stresses, i.e. UV exposure and mechanical stretching, before analyzing p53. Cells subjected to UVB irradiation (10–30 mJ/cm^2^) showed a trend of elevated p53/p21^WAF1/CIP1^ after 1d. This effect was enhanced markedly in Dsg3 depleted cells, indicating Dsg3’s ability to antagonize UV induced p53 expression (Figs. 1e, 3a). Reduction in cell cycle regulators PCNA and Cyclin A was demonstrated by Western blotting analysis in knockdown cells in both the presence and absence of UV (Fig. 3d). Moreover, Bax immunostaining indicated enhanced signals in both the cytoplasmic and nuclear compartments in RNAi treated cells (Fig. S4a). Consistently, overexpression of Dsg3 resulted in suppression of p53/p21^WAF1/CIP1^ after UV (Fig. S2a). Moreover, these cells were highly resistant to UV-induced cell death relative to control cells. Similar findings were made in A2780 and HCT116 (wtp53) (Fig. S2b). Additionally, cells treated with genotoxic drugs, such as actinomycin D (Act D) or mitomycin C (MMC), also showed a similar effect with strong induction of p53/p21^WAF1/CIP1^ in Dsg3 depleted cells compared to cells without knockdown (Fig. 3b). These data suggest that the expression of Dsg3 protects cells against various environmental insults by dampening the p53 response. This hypothesis was supported by additional mechanical loading experiments in which siRNA treated cells were challenged with equiaxial cyclic strain (FX-5000: 1 Hz, 20%) for 4 h. Lysates extracted either immediately after strain (0 h) or 2 and 24 h later, respectively, followed by Western blotting analysis. It showed increased p53/p21^WAF1/CIP1^/Bax in Dsg3 knockdown cells, particularly at 0 h and 2 h in post-strained cells (Fig. 3c). Thus the loss of Dsg3 affected p53 stabilization in response to mechanical stress reinforcing its ability to counterbalance p53 response to mechanical stress.

To explore the upstream components involved in p53 activation in our knockdown system, we analyzed total and phosphorylated protein expression of ATM serine/threonine protein kinase, as well as one of its targets CHK2, which is activated by DNA double-strand breaks^33^. Cells were subjected to UV or no UV exposure for 1 day before Western blotting analysis (Fig. 3e). Although the effects were modest both phospho-ATM (pATM) and -CHK2 (pCHK2) showed elevated levels as well as total p53/phospho-p53-S20 in Dsg3 depleted cells, with further enhancement by UVB. Both total proteins also showed a similar expression pattern with increased levels in knockdown cells compared to controls, regardless of UV. These results suggest that the DNA double-strand break induced activation of pATM is involved in the upregulation of the p53 pathway in Dsg3 depleted cells. To confirm the presence of DNA double-strand breaks in these cells we performed immunofluorescence for 53BP1 and measured the nuclear foci in both control and Dsg3 depleted cells with and without UVB. Increased number of 53BP1 foci indicated enhanced DNA double-strand breaks, in particular in Dsg3 depleted cells exposed to UVB which were significantly different from scrambled siRNA controls (Fig. 3f). Finally, cells were treated in the presence and absence of KU55933 (ATM inhibitor) and SB203580 (p38 MAPK inhibitor) and both drugs inhibited the enhanced CHK2 phosphorylation in Dsg3 depleted cells relative to corresponding scrambled siRNA controls in parallel with a strong reduction in total p53/phospho-p53-S20 (Fig. 3g, h) and this was particularly marked in the case of SB203580 (Fig. 3h right panel). These findings suggest that, in the presence of DNA damage, Dsg3 depletion further potentiates DNA double-strand breaks, which are involved in activation of ATM and its downstream targets CHK2 and p53, leading to cell cycle arrest and pre-apoptosis.

Again, the knockdown studies for desmoplakin and E-cadherin were performed in conjunction with UV but no comparable results were obtained, albeit the UV exposure induced p53 (Fig. 1g, h). Only a small reduction of p53/p21^WAF1/CIP1^/Bax was detected in E-cadherin knockdown. No apparent changes of p53 were shown in desmoplakin knockdown while it had no effect on p21^WAF1/CIP1^ and only caused a marginal increase in Bax. Collectively, these data suggest that the regulation of p53 by Dsg3 likely is independent of desmoplakin and E-cadherin, implying that this pathway may be mediated by extra-desmosomal Dsg3^17^.

### Increased p53 expression and activity in Dsg3^−/−^ mouse skin in vivo

**Fig. 4:**
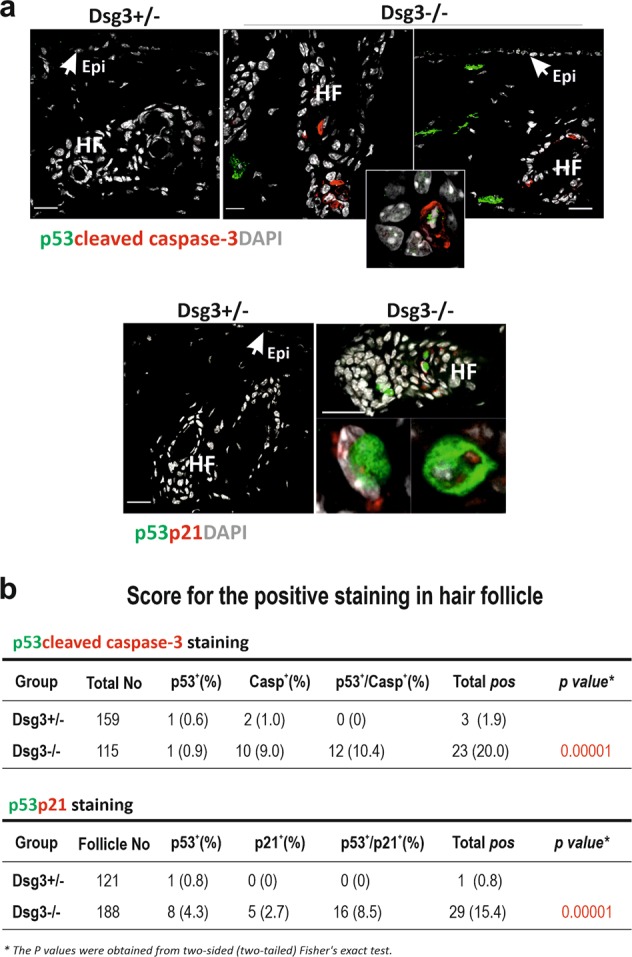
Increased expression of p53, p21^WAF1/CIP1^ and cleaved caspase-3 is observed in the back skin of Dsg3 knockout mice. **a** Immunofluorescent staining in the back skin of Dsg3^−/−^ and Dsg3^+/−^ (heterozygous littermate) mice showed elevated signals for the indicated proteins in the hair follicles of Dsg3^−/−^ mice compared to heterozygous littermate, though no positive staining was observed in the epidermis (*n* = 2 mice per group, aged 8–12 weeks). Some fibroblasts in the dermis were also shown positive staining of p53. Epi: epidermis, HF: hair follicle. The inset in the top right panel highlight cells with double positive staining for p53 and active caspase-3 in Dsg3 null skin. Scale bar, 20 µm. **b** Tables summarize the scores of positive hair follicle staining for p53/active caspase-3 and p53/p21^WAF1/CIP1^, respectively. Each hair follicle containing one or more positively stained keratinocytes was scored positive

Having confirmed that Dsg3 negatively regulates p53 in keratinocytes, we then asked whether alteration of this pathway is detectable in Dsg3 knockout mice. Mice with targeted ablation of Dsg3 exhibit runting and wave-pattern hair loss, accompanied by oral and skin lesions, after weaning^34,35^. Increased expression of p53/p21^WAF1/CIP1^/cleaved caspase-3 was detected in Dsg3^−/−^ hair follicles in dorsal skin samples from such mice, but not in Dsg3^+/−^ littermates (Fig. 4). This result confirmed that Dsg3 expression is associated with the prevention of p53 activation in mouse skin in vivo.

### Enhanced p53 expression in PV and in keratinocyte cultures treated with PV sera as well as a characterized pathogenic antibody

**Fig. 5:**
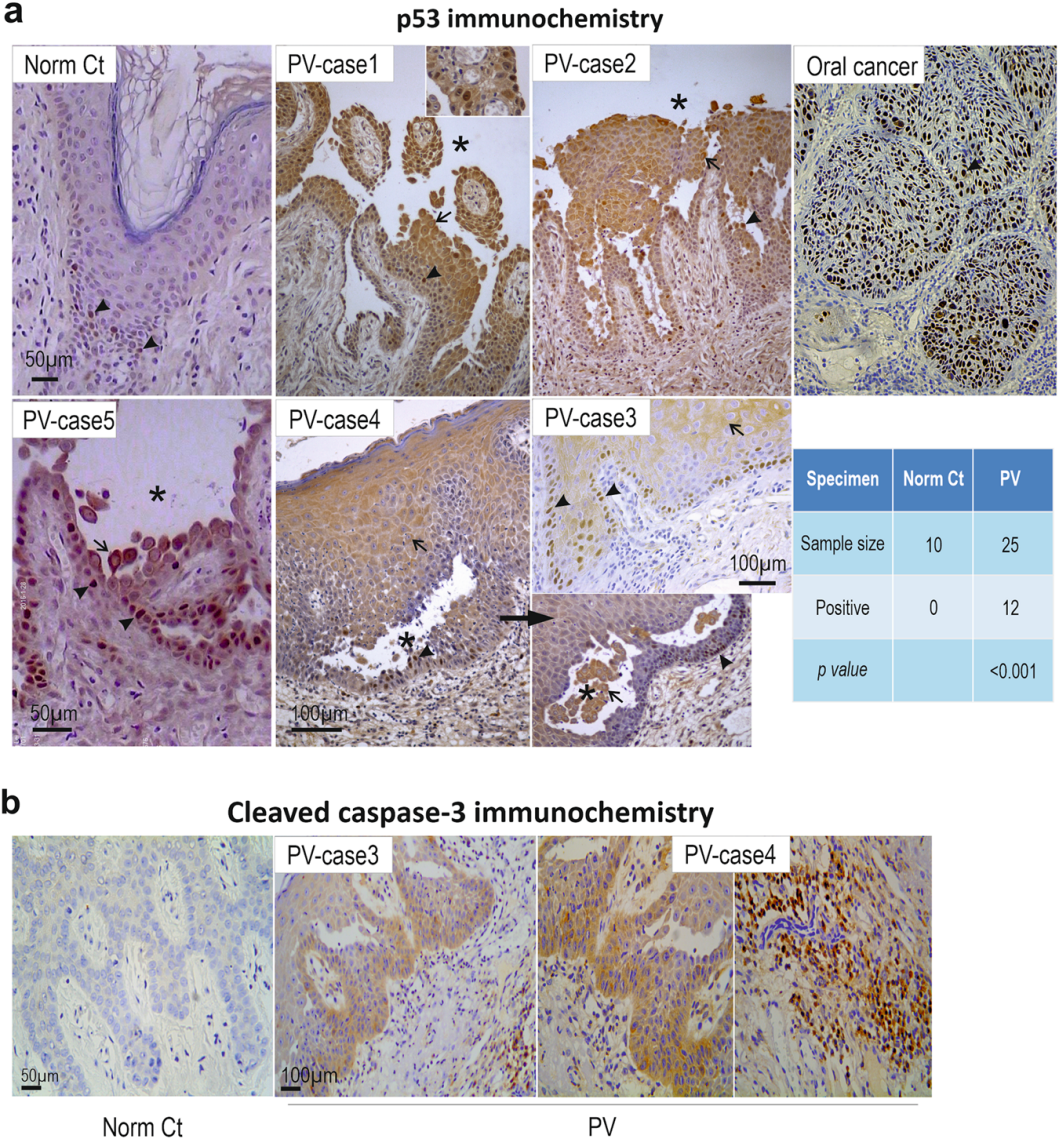
Enhanced p53 and cleaved Caspase-3 expression is shown in clinical PV patient samples and also in keratinocyte cultures treated with PV sera. p53 **a** and cleaved Caspase-3 **b** immunohistochemistry in oral mucous tissues from PV patients. Significantly enhanced p53 staining was detected in 48% of patients (arrowheads indicate positive nuclear staining whereas arrows indicate predominant cytoplasmic staining), compared to normal controls. Oral mucous cancer was used as positive control here. Asterisks indicate the areas of the blisters. Positive staining of active caspase-3 was also detected in PV patients with the positive p53 staining in oral mucous tissues, especially in the basal and immediate suprabasal layers of stratified squamous epithelium. The positive staining was also detected in cells located in sub-mucous connective tissue (right)

To explore whether our identified pathway is operative in PV, we performed immunohistochemistry for p53 in oral tissue biopsies from 25 patients and found a remarkable increase of p53, in both cytoplasm and nucleus across almost the entire stratified epithelial layer, in 12 PV cases (~50%), especially in cells surrounding or in the clusters within blisters. Normal samples showed only a few p53 nuclear-positive cells located in the basal and suprabasal layers (Fig. 5a). Cells immunopositive for cleaved caspase-3 in PV were also positive for p53, indicating activation of the p53 pathway in PV (Fig. 5b). Notably, positive staining for both proteins was observed in non-lesional areas in PV (Case-3 in Fig. 5). These results are indicative of alterations of the Dsg3-p53 pathway in PV that lead to caspase-3 activation as reported previously^36^.

**Fig. 6:**
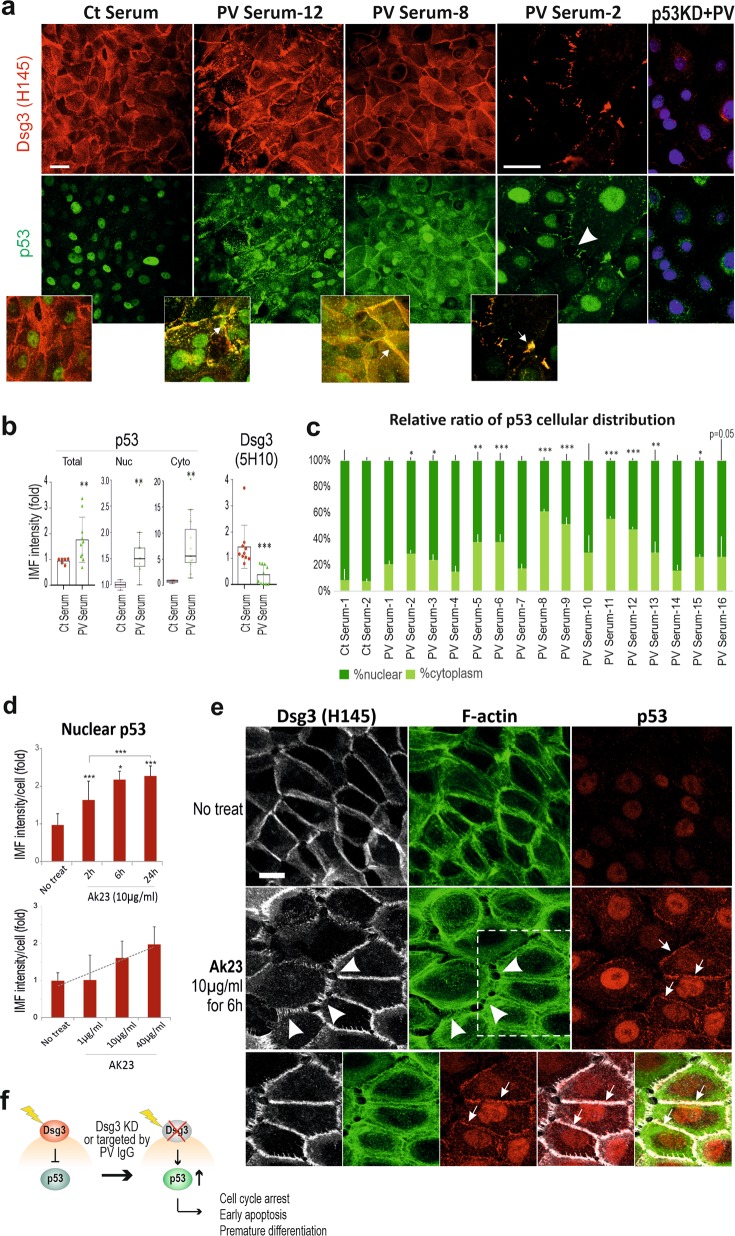
Altered p53 expression and distribution were detected in keratinocyte cultures treated with PV sera and pathogenic antibody. **a** Confocal microscopy of NTERTs treated with the PV sera, dual labeled for Dsg3 and p53. Cells were seeded at confluent densities in KGM for overnight before being treated with PV sera (at 40% concentration in KGM) from a different cohort of PV patients (*n* = 17), for 24 h. Disruption or depletion of Dsg3 at the plasma membrane accompanied with marked increases in p53 was observed in PV serum-treated cells that were abolished by p53 knockdown, compared to controls exposed to sera of healthy individuals that displayed, predominantly, nuclear p53 signals. Of note, p53 also showed distribution at the membrane where it colocalized with the fragmented Dsg3 (arrows in the inserts). Additional data for p53 knockdown was shown in Supplementary material Fig. S5. The image magnification in PV serum-2 was doubled, relative to other panels, to highlight the disruption of junctions and peripheral distribution of p53. **b** Scatter and whisker plots of Dsg3 and p53 cellular and subcellular expression (*n* = 16 for PV serum samples, *n* = 6 for control samples). Student *t*-test and the Wilcoxon–Mann–Whitney Rank Test were used for statistically significant analysis here and gave similar results. **c** The relative ratio of p53 nuclear versus cytoplasmic cellular distribution in controls and 16 PV sera treated NTERTs. **d** Treatment of NTERTs with the pathogenic monoclonal antibody AK23 targeting Dsg3 N-terminus, caused p53 induction, in a time and dose-dependent manner (*n* = 11 fields per condition, pooled from 2 independent experiments). For the dose-response experiment, cells were treated with AK23 for 6 h. **e** Confocal images of cells with triple staining, treated in the presence and absence of AK23. Disruption of F-actin along with Dsg3 (arrowheads) was readily detectable in cells exposed to AK23. Increase p53 expression was detected predominantly in the nucleus and also was observed at the plasma membrane where it showed colocalization with Dsg3 and F-actin (arrows) in cells treated with AK23. The membrane distribution of p53 was not detectable in control cells. The protein colocalization of the dotted line box is shown at the bottom. (*p < 0.05, **p < 0.01, ***p < 0.001). Scale bar, 10 µm. **f** Schematic model of Dsg3 in the suppression of p53 in keratinocyte response to stresses. Disruption of Dsg3 by RNAi or PV autoantibodies evokes p53 induction

Since activation of p53 occurs in other diseases as described above, it is important to determine the specificity of this Dsg3-p53 pathway in PV. To evaluate our in vivo finding in PV, next, in vitro studies were performed with PV sera collected from a different cohort of 17 patients, and more specifically, with AK23, a well-characterized pathogenic monoclonal antibody targeting the adhesion site at Dsg3 N-terminus^37^. Confluent cells were treated with PV sera or AK23 (40% PV sera, 1–100 µg/ml AK23) before immunostaining for p53/Dsg3 (Fig. 6a–e). In controls treated with normal sera, nuclear p53 was predominant, with limited cytoplasmic staining. In contrast, cells treated with PV sera (for 24 h) exhibited an augmentation of both nuclear and cytoplasmic p53 (Fig. 6a–c). In some samples, membranous and cytoplasmic staining was evident. Intriguingly, the membrane distribution of p53 showed co-localization with Dsg3 where there was severe membrane disruption (Fig. 6a arrows in the inserts). The enhanced cytoplasmic p53 in PV serum-treated cells may indicate the enhanced protein synthesis (Fig. 6c). In parallel, treatment with AK23 mirrored increased nuclear p53, in a time and dose-dependent manner (Fig. 6d, e). As expected, disruption of junctions was apparent in cells with membrane distribution of p53 (arrows in Fig. 6e). Some difference in p53 staining was observed between AK23 and PV serum-treated cells suggesting variations between monoclonal antibody and patient sera with polyclonal antibodies. The specificity of enhanced p53, induced by PV sera, was confirmed by p53 knockdown experiments where cells were transfected with p53 siRNA alongside with control siRNA for 1d before treated with PV or control sera. p53 knockdown almost completely abolished p53 signals in control of serum-treated cells (Fig. S3). However, in the PV serum treated samples, although some non-specific background in p53 staining was shown in cases, p53 knockdown rendered significant reduction of p53 (Fig. 6a right panel, Figure S3). Additionally, Bax staining showed enhanced cytoplasmic signals in cells treated with PV sera (Fig. S4b). For Dsg3 staining, two Dsg3 antibodies were used. While Dsg3 staining with rabbit antibody (H145) that binds to its C-terminus exhibited broad variations, another mouse antibody (5H10) that binds the N-terminus at the extracellular domain of Dsg3 showed marked depletion of Dsg3 from the surface in cells treated with PV sera (Fig. 6a, b). Drastic disruption/reduction of Dsg3 at the junctions was detected with H145 in cells treated with PV sera and to a lesser extent, with AK23 (arrowhead in Fig. 6a, e). Some PV sera samples even showed a marked increase accompanied with pronounced Dsg3 disruption at the junctions and its aggregates in the cytoplasm (PV serum-12, Fig. 6a). Taken together, these in vitro findings demonstrate that treatment of keratinocytes with PV autoantibodies and pathogenic antibody evoked marked disruption/depletion of Dsg3 from the plasma membrane, leading to induction of p53 and activation.

## Discussion

p53, a “guardian of the genome”, is a central player in cell responses to environmental stress^23,29^. Here we provide the first evidence that Dsg3 acts as an anti-stress protein by restricting p53 responses to stress signals in keratinocytes (Fig. 6f). Knockdown of Dsg3 in vitro or its ablation in vivo caused elevated expression/stabilization of p53, coupled with decreased PCNA and Cyclin A, and increased activation of caspase-3, indicative of early apoptosis^31,36^. Our in vitro studies suggest an involvement of at least ATM and CHK2 activation upstream of p53 upregulation, following Dsg3 depletion. This effect was more pronounced in cells exposed to various stresses. Conversely, overexpression of Dsg3 resulted in the opposite effect with marked suppression of p53. Finally, we showed that this pathway seemed to be altered in PV, at least in a subset of patients, suggesting that the antibodies which mediated Dsg3 disruption induces p53 activation. This finding underscores the possibility that failure in this anti-stress pathway contributes to PV acantholysis.

PV is caused by autoantibodies targeting Dsgs that lead to defects in cell–cell adhesion, however, the precise molecular mechanism is still not fully understood. Previous studies indicated that apoptosis and activation of caspases are involved in PV pathogenesis with the hypothesis that an activated death signaling could be the underlying mechanism in PV-IgG induced acantholysis^38–41^. Increased FasL/FasR were detected in keratinocyte and skin organ cultures treated with PV-IgG^39–41^. In addition, there is evidence suggesting that blistering is associated with surface receptors other than Dsgs^16,17,42^. Thus, the direct link between Dsg3 and the p53 pathway is unclear. In this study, we provide evidence of a potential link between Dsg3 and p53, indicating that disruption of Dsg3 by PV IgG may cause p53 induction leading to caspase-3 activation. Our in vitro study demonstrated that Dsg3 depletion causes enhanced p53 with elevated expression of its targets p21^WAF1/CIP1^, resulting in a reduction of PCNA and Cyclin A coupled with elevated Bax/cleaved (activated) caspase-3. We also showed that such responses were accelerated when cells were exposed to stresses that trigger a p53 activation. These in vitro findings were supported by an in vivo study in Dsg3 null mice, as well as by the gain-of-function studies that caused marked suppression of p53 and its transcription activity. These findings collectively support the hypothesis that Dsg3 dampens the stress response pathway by negatively regulating p53. Notably, we showed that this pathway is altered in ~50% PV patient samples with enhanced p53 and caspase-3 that was not only found in cells surrounding the blisters but also in pro-lesions regions, indicating that activation of the p53 pathway occurs early prior to the event of pemphigus acantholysis. The apparent heterogeneity of p53 elevation in PV patients could well reflect variations of clinical activities/treatment status of the disease and/or be related to the transient response of p53 to cellular stress. It is well-known that drugs which caused DNA damage initially induce high levels of p53, as well as p21^WAF1/CIP1^, but these changes decline over time^43^. It is worth noting that the specificity of p53 induction caused by antibody targeting Dsg3 was verified by our in vitro studies with PV sera (samples 17 cases) and a well-characterized specific pathogenic monoclonal antibody, AK23 targeting the Dsg3 adhesion site. Thus, our findings in PV may indicate a specific p53 induction associated with the PV IgG induced Dsg3 disturbance since treatment of cells with AK23 caused augmented p53 in a time and dose-dependent manners. Taken together, these results suggest that activation of the Dsg3-p53 pathway may contribute, at least in part, to PV pathology^36,39,40^.

Activation of p53 also occurs in other chronic or inflammatory conditions including LP^26–28^, psoriasis^24,25^ and Rheumatoid arthritis^44^ in which the role of Dsg3 has not yet been defined. Thus, disruption of Dsg3 may be one mechanism by which p53 can be activated in human skin diseases. As p53 is a key factor in cell cycle control, differentiation and apoptosis as well as a valuable biomarker for prediction of malignant transformation, it is not surprising to see the altered p53 expression in other conditions although the molecular basis underlying the p53 activation may differ. Elevated p53 in psoriasis was thought to be associated with cell proliferation^24,25^ whereas its detection in LP might be due to p53 gene mutation^26–28^. Furthermore, an activated p53 pathway can elicit pro-apoptotic/apoptotic events through multiple mechanisms depending on the location and levels of its expression. The cytoplasmic p53 can mediate apoptosis by directly interacting with Bcl-2 family members^45^, while nuclear p53 can bind to DNA and activate pro-apoptotic gene expression, which ultimately contributes to disease pathology.

The finding of increased caspase-3 in cells with Dsg3 knockdown may indicate early apoptosis, however, our FACS analysis for Annexin V did not detect any evident apoptosis in Dsg3 knockdown cells. A recent study has highlighted that a transient, modulated activation of caspase-3 is triggered by antibody-mediated Dsg3 disruption in PV, but this event is uncoupled from the classical apoptotic pathways^36^. Nevertheless, active caspase-3 in PV seems to be a consensus finding and inhibition of caspase-3 has been shown to be effective in preventing blister formation in both in vitro and in vivo PV models. Thus, the anti-Dsg3 antibody mediated activation of p53 and its regulated target caspase-3 could well be the key factor to cause PV acantholysis. In support of this notion, activation of caspase-3 has been reported to be a causative factor for the rapid disruption of tight junctions in endothelial cells^46^. Furthermore, the staurosporine induces activation of caspase-3 is accompanied by disturbance of adherens junctions accompanied by a significant increase in cell permeability which can be inhibited by pretreatment with caspase-3 specific inhibitor^47^.

PV is a complex autoimmune disease with Dsg3 being a central player in pemphigus acantholysis that likely is triggered by a collection of signaling pathways, including Src, p38 MAPK, EGFR, c-Myc, and Rho GTPases, downstream of PV-IgG targeting Dsg3 disruption^4,17^. We now report a potential involvement of abnormal p53 activation in PV caused by PV IgG targeting Dsg3 which acts as an anti-stress protein in counterbalancing p53 in the maintenance of normal epithelial homeostasis.

## Materials and methods

### Cell lines, animal and clinical patient oral mucosal samples

Various epithelial cell lines derived from skin and other tissues were used in the study, i.e. NTERT immortalized skin keratinocytes (wild-type p53: wtp53) maintained in keratinocytes serum-free medium (KSFM) (17005042, Thermo Scientific); T8 cutaneous squamous cell carcinoma cell line with a frameshift mutation at amino acid 91 of *TP53* resulting in a truncated protein and making it essentially p53 null (gift from Prof. Catherine Harwood), and they were cultured in complete keratinocyte growth medium KGM containing Dulbecco’s Modified Eagle Medium (DMEM) (12–604F, Lonza):Ham’s 12 (11765054, Thermo Scientific) in the ratio of 3:1 supplemented with 10% fetal calf serum (FCS) (Biosera), epidermal growth factor (EGF) (13247-051, Invitrogen), Insulin human solution (19278, Sigma), cholera toxin (C8052, Sigma), and hydrocortisone (H4001, Sigma). MDCK (Madin Darby canine kidney) cells (wt p53) are the simple epithelial cell line, which is derived from canine kidney tubule epithelium; A431 cell line (mutant p53-R273H) is derived from vulva squamous cell carcinoma; A2780 ovarian cancer cell line (wt p53) and HCT116 colorectal carcinoma cell line (wt p53). All these cell lines were maintained in DMEM (12–604 F, Lonza) supplemented with 10% FCS (Biosera, UK). Due to the low levels of endogenous Dsg3 expression, these cell lines were used for the gain-of-function studies by transduction of retroviral construct pBABE-hDsg3.myc along with the empty vector control^3,48^ namely FL Dsg3 and Vect Ct cells, respectively^3^. Cells were incubated at 37 °C in a humidified atmosphere of 95% air and 5% CO_2_. The medium was changed on alternate days and cells were subjected to subculture routinely once they reached to about 70–80% confluence.

Mouse back skin samples from Dsg3 null (Dsg3^−/−^) and heterozygous control littermates (Dsg3^+/−^) were obtained, as described previously^49^. PV sera (anonymous, 17 cases) were received from our collaborator based in First Department of Dermatovenerology, St. Anne’s Faculty Hospital, Brno, Czech Republic, and oral tissue samples of PV patients (25 PV cases and 10 normal healthy tissue controls as well as 3 cancer patient samples) were obtained from our collaborator in Guiyang Medical University, China; all with informed patient consent and ethical approval.

### Antibodies

The following mouse (m) and rabbit (r) monoclonal/polyclonal antibodies (Abs) were used: Dsg3 mAb against the N-terminus (5H10) (sc-23912, Santa Cruz); Dsg3 rAb against the C- terminus (H145) (sc-20116, Santa Cruz); p53 mAb (DO-1) (ab1101, Abcam); p53 rAb (C-19) (sc-1311-R, Santa Cruz); MDM2 rAb (EP16627) (ab178938, Abcam); phospho MDM2 rAb (S166) (ab131355, Abcam); p21^WAF1/CIP1^ rAb (C-19) (sc-397, Santa Cruz); Bax mAb (sc-20067, Cell Signaling); Caspase3 rAb (clone C92-605, RUO) (14C10, BD Biosciences); Caspase3 rAb (9662 S, Cell Signaling); active Caspase3 rAb (ab49822, Abcam); Desmoplakin rAb (sc-33555, Santa Cruz); Plakoglobin mAb (PG51, Progen); Dsc2 rAb (610120, Progen); Dsg2 mAb (33-3D) was kindly received from Prof. David Garrod; E-Cadherin mAb (HECD-1) (ab1416, Abcam); H-432, rabbit Ab to Cyclin A (sc-751, Santa Cruz); PC10, mouse Ab to PCNA (sc-56, Santa Cruz); Glyceraldehyde-3-phosphate dehydrogenase (GAPDH) rAb (14c10, Cell Signaling); HSC70 mAb (B6:sc-7298, Santa Cruz); β-actin mAb (8H10D10, Cell Signaling); anti-53BP1 (05–726, Upstate); anti-ATM (phospho S1981) (ab81292, Abcam); anti-ATM (ab32420, Abcam); anti-CHK2 (phospho T68) (ab32148, Abcam); anti-CHK2 (ab109413, Abcam). The anti-ATM antibodies were validated on Western blots of a range of normal and neoplastic oral keratinocytes lines and ATM-deficient human epidermal keratinocytes from an Altaxia Telangiectasia patient, the last of which showed no ATM protein expression (K.Ng and E.K.Parkinson - manuscript in preparation).

### Treatments with ultraviolet (UV) B, drugs, and mechanical stretching

The siRNA treated cells were seeded at confluence densities in 6-well plates before irradiation of UVB (10–30 mj/cm^2^) using a CL-1000 Ultraviolet Crosslinker (Ultra-Violet Products, CA) or treatment with actinomycin D (Act-D, 5 nM) and mitomycin C (MMC, 5 ug/ml) for 24 h, respectively. Protein lysates were extracted for Western blotting analysis.

The regimen for the cyclic strain was adapted from a previous publication^50^. Briefly, cells were plated at confluent densities and grown for 1–2 days on collagen-coated BioFlex 6-well plates (Flexcell® International Corporation) prior to equiaxial cyclic stretching (20% amplitude with 1 Hz, FX-5000 Tension System (Flexcell International, Burlington, NC) for 4 h). Control cells were seeded in the same plates without strain. Lysates were extracted either immediately after strain or transferred to the static state in an incubator and harvested later for the indicated time points.

The details of siRNA/plasmid transfection/transduction, immunofluorescence, immunohistochemistry in PV specimens, nuclear extraction, Western blotting, luciferase assay, FACS based Cell Viability-Caspase-3 assay and RT-qPCR, etc. were described in Supplementary Materials.

### Statistical analysis

Statistical differences between control and test groups were analyzed using unpaired, 2-tailed Student *t*-test in most cases. For some experiments, the data were analyzed by the Wilcoxon–Mann–Whitney Rank Test. Data are presented as mean ± s.d. unless otherwise indicated. Two-sided Fisher’s exact test was used for the comparison of the positive hair follicle scoring in mice. Chi-Square statistic was used for obtaining the *p* values in the comparison between PV patient samples and controls. P values of less than 0.05 were considered statistically significant. Experiments were repeated at least three times. The microscopic images were acquired in >4–6 arbitrary fields per sample. For Western blotting analysis, lysates were collected from three biologically independent replicates. Wherever possible, the comparison between control and test groups was normalized against the control and expressed as a fold change relative to controls (set as 1).

## Supplementary information

Supplemental materials

Fig. S1. Keratinocyte differentiation marker showed an inverse relationship with Dsg3 expression levels

Fig. S2. Overexpression of Dsg3 in various cell lines protects cells from the UV induced cell death

Fig. S3. p53 knockdown results in a significant reduction of p53 staining signals in cells treated with PV sera

Fig. S4. Both Dsg3 knockdown and PV sera treatment cause increased Bax expression in cells, in the cytoplasm and/or nucleus

## References

[1] Brown L, Wan H (2015). Desmoglein 3: a help or a hindrance in cancer progression?. Cancers.

[2] Mannan T (2011). RNAi-mediated inhibition of the desmosomal cadherin (desmoglein 3) impairs epithelial cell proliferation. Cell Prolif..

[3] Tsang SM (2010). Desmoglein 3, via an interaction with E-cadherin, is associated with activation of Src. PLoS. ONE.

[4] Tsang SM (2012). Non-junctional human desmoglein 3 acts as an upstream regulator of Src in E-cadherin adhesion, a pathway possibly involved in the pathogenesis of pemphigus vulgaris. J. Pathol..

[5] Tsang SM (2012). Desmoglein 3 acting as an upstream regulator of Rho GTPases, Rac-1/Cdc42 in the regulation of actin organisation and dynamics. Exp. Cell Res..

[6] Rotzer V (2016). Desmoglein 3-dependent signaling regulates keratinocyte migration and wound healing. J. Invest Dermatol..

[7] Chen YJ (2013). DSG3 facilitates cancer cell growth and invasion through the DSG3-plakoglobin-TCF/LEF-Myc/cyclin D1/MMP signaling pathway. PLoS ONE.

[8] Amagai M (1996). Pemphigus vulgaris antigen (desmoglein 3) is localized in the lower epidermis, the site of blister formation in patients. J. Invest Dermatol..

[9] Teh MT (2011). A molecular study of desmosomes identifies a desmoglein isoform switch in head and neck squamous cell carcinoma. J. Oral. Pathol. Med..

[10] Amagai M, Klaus-Kovtun V, Stanley JR (1991). Autoantibodies against a novel epithelial cadherin in pemphigus vulgaris, a disease of cell adhesion. Cell.

[11] Kitajima Y (2014). 150(th) anniversary series: Desmosomes and autoimmune disease, perspective of dynamic desmosome remodeling and its impairments in pemphigus. Cell Commun. Adhes..

[12] Calkins CC (2006). Desmoglein endocytosis and desmosome disassembly are coordinated responses to pemphigus autoantibodies. J. Biol. Chem..

[13] Delva E (2008). Pemphigus vulgaris IgG-induced desmoglein-3 endocytosis and desmosomal disassembly are mediated by a clathrin- and dynamin-independent mechanism. J. Biol. Chem..

[14] Lanza A (2006). How does acantholysis occur in pemphigus vulgaris: a critical review. J. Cutan. Pathol..

[15] Amagai M (2006). Are desmoglein autoantibodies essential for the immunopathogenesis of pemphigus vulgaris, or just “witnesses of disease”?. Exp. Dermatol..

[16] Grando SA (2009). Apoptolysis: a novel mechanism of skin blistering in pemphigus vulgaris linking the apoptotic pathways to basal cell shrinkage and suprabasal acantholysis. Exp. Dermatol.

[17] Spindler V (2018). Mechanisms causing loss of keratinocyte cohesion in pemphigus. J. Invest Dermatol..

[18] Brown L (2014). Desmoglein 3 promotes cancer cell migration and invasion by regulating activator protein 1 and protein kinase C-dependent-Ezrin activation. Oncogene.

[19] Chen YJ (2007). DSG3 is overexpressed in head neck cancer and is a potential molecular target for inhibition of oncogenesis. Oncogene.

[20] Kennedy, B. G. & J. E. Lever. Regulation of Na+,K+-ATPase activity in MDCK kidney epithelial cell cultures: role of growth state, cyclic AMP, and chemical inducers of dome formation and differentiation. *J. Cell Physiol*. **121**, 51–63 (1984). 10.1002/jcp.1041210108 6090479

[21] Leighton, J. et al. A cell line derived from normal dog kidney (MDCK) exhibiting qualities of papillary adenocarcinoma and of renal tubular epithelium. *Cancer*. **26**, 1022–1028 (1970). 10.1002/1097-0142(197011)26:5<1022::aid-cncr2820260509>3.0.co;2-m 4248968

[22] Oberleithner, H., Vogel, U. & Kersting, U. Madin-Darby canine kidney cells. I. Aldosterone-induced domes and their evaluation as a model system. *Pflugers Arch*. **416**, 526–532 (1990). 10.1007/BF00382685 2235294

[23] Vousden KH, Lu X (2002). Live or let die: the cell’s response to p53. Nat. Rev. Cancer.

[24] Batinac T (2007). Expression of Bcl-2 family proteins in psoriasis. Croat. Med. J..

[25] Kim SA (2018). Differential expression of cyclin D1, Ki67, pRb, and p53 in psoriatic skin lesions and normal skin. Mol. Med. Rep..

[26] Shiva A (2018). Immunohistochemical study of p53 expression in patients with erosive and non-erosive oral lichen planus. J. Dent..

[27] Hadzi-Mihailovic M (2017). Expression and role of p53 in oral lichen planus patients. J. Buon..

[28] Acay RR (2006). Evaluation of proliferative potential in oral lichen planus and oral lichenoid lesions using immunohistochemical expression of p53 and Ki67. Oral. Oncol..

[29] Purvis JE (2012). p53 dynamics control cell fate. Science.

[30] Kubbutat MH, Jones SN, Vousden KH (1997). Regulation of p53 stability by Mdm2. Nature.

[31] Hague A (2004). Caspase-3 expression is reduced, in the absence of cleavage, in terminally differentiated normal oral epithelium but is increased in oral squamous cell carcinomas and correlates with tumour stage. J. Pathol..

[32] Lee HL (2018). Simultaneous flow cytometric immunophenotyping of necroptosis, apoptosis and RIP1-dependent apoptosis. Methods.

[33] Marechal, A. & L. Zou. DNA damage sensing by the ATM and ATR kinases. *Cold Spring Harb. Perspect. Biol*. **5**, a012716 (2013). 10.1101/cshperspect.a012716 PMC3753707 24003211

[34] Koch PJ (1998). Desmoglein 3 anchors telogen hair in the follicle. J. Cell Sci..

[35] Koch PJ (1997). Targeted disruption of the pemphigus vulgaris antigen (desmoglein 3) gene in mice causes loss of keratinocyte cell adhesion with a phenotype similar to pemphigus vulgaris. J. Cell Biol..

[36] Luyet C (2015). Preclinical studies identify non-apoptotic low-level caspase-3 as therapeutic target in pemphigus vulgaris. PLoS. ONE.

[37] Tsunoda K (2003). Induction of pemphigus phenotype by a mouse monoclonal antibody against the amino-terminal adhesive interface of desmoglein 3. J. Immunol..

[38] Gniadecki R (1998). Relationship between keratinocyte adhesion and death: anoikis in acantholytic diseases. Arch. Dermatol. Res..

[39] Pelacho B (2004). Pemphigus vulgaris autoantibodies induce apoptosis in HaCaT keratinocytes. FEBS Lett..

[40] Puviani M (2003). Fas ligand in pemphigus sera induces keratinocyte apoptosis through the activation of caspase-8. J. Invest Dermatol..

[41] Wang X (2004). Possible apoptotic mechanism in epidermal cell acantholysis induced by pemphigus vulgaris autoimmunoglobulins. Apoptosis.

[42] Grando SA (2012). Pemphigus autoimmunity: hypotheses and realities. Autoimmunity.

[43] Robles SJ, Adami GR (1998). Agents that cause DNA double strand breaks lead to p16INK4a enrichment and the premature senescence of normal fibroblasts. Oncogene.

[44] Zhang T (2016). p53 predominantly regulates IL-6 production and suppresses synovial inflammation in fibroblast-like synoviocytes and adjuvant-induced arthritis. Arthritis Res. Ther..

[45] Geng Y (2010). Cytoplasmic p53 and activated Bax regulate p53-dependent, transcription-independent neural precursor cell apoptosis. J. Histochem. Cytochem..

[46] Zehendner CM (2011). Caspase-3 contributes to ZO-1 and Cl-5 tight-junction disruption in rapid anoxic neurovascular unit damage. PLoS ONE.

[47] Sawant DA (2013). Microvascular endothelial cell hyperpermeability induced by endogenous caspase 3 activator staurosporine. J. Trauma Acute. Care Surg..

[48] Moftah, H. et al. Desmoglein 3 regulates membrane trafficking of cadherins, an implication in cell-cell adhesion. C*ell Adh. Migr.* **11**, 1–22 (2016). 10.1080/19336918.2016.1195942 PMC5479455 27254775

[49] Hunefeld C (2018). Bone marrow-derived stem cells migrate into intraepidermal skin defects of a desmoglein-3 knockout mouse model but preserve their mesodermal differentiation. J. Invest. Dermatol..

[50] Russell D (2004). Mechanical stress induces profound remodelling of keratin filaments and cell junctions in epidermolysis bullosa simplex keratinocytes. J. Cell Sci..

