# Supplementary material for: The desmosomal cadherin desmoglein-3 acts as a keratinocyte anti-stress protein via suppression of p53
Source: Cell Death Dis. 2019 Oct 3;10(10):750. doi: 10.1038/s41419-019-1988-0 (PMC6776551; doi:10.1038/s41419-019-1988-0)
Supplement: Supplementary file 1 — Supplemental materials [file 41419_2019_1988_MOESM1_ESM.docx]

Rehman et al. **- Supplementary material**

**The desmosomal cadherin desmoglein-3 acts as an anti-stress protein via suppression of p53 in keratinocytes.**

**Materials and methods**

**siRNA and plasmid transfection and transduction**

All transient siRNA transfections at 100 nM final concentration were conducted either by following the protocols described previously^1-3^ or using DharmaFECT 1 (T-2001-02, Dharmacon) following the manufacturer’s instructions. The self-designed siRNA (AAATGCCACAGATGCAGATGA) corresponding to nucleotides 620–640 of human Dsg3 mRNA (Accession No: NM_001944) and a scrambled control sequence (AACGATGATACATGACACGAG) were synthesized by Dharmacon (USA). Other siRNA sequences for desmoplakin^4^, E-cadherin (ON-TARGETplus SMARTpool L-003877-00-0005), p53 (GAAAUUUGCGUGUGGAGUA) were purchased from Dharmacon. NTERT cells expressing high levels of endogenous Dsg3 were used for the loss-of-function study. In brief, 2x105 cells were seeded into a 6-well plate and allowed to grow overnight. Cells were then transfected with either scrambled or specific siRNA at a final concentration of 100 nM in Opti-MEM I (31985-062, Gibco) using oligofectamine transfection reagent (12252011, Invitrogen)/ DharmaFECT 1. Transfection was performed for 4 hours before addition of FCS at a final concentration of 10%. Cells were grown overnight before being harvested with 0.25% trypsin/EDTA (T3924, Sigma) and re-plated at the densities according to the experiments.

Other cell lines including T8, HCT116, A2780, MDCK and A431 cells expressing low levels of endogenous Dsg3 were used for the gain-of-function approach. The transduction of pBABE-puro vector control and *hDsg3.myc* was generated following procedures as described previously^5,6^. For transient transfection of plasmid DNA of pcDNA3.1-p53 WT, along with the empty vector control plasmid, cells were seeded in a 6-well plate at a density of 2x105. On the following day, the transfection cocktail was prepared by mixing 2 ug of the plasmid with 3x of Fugene HD reagent (E2311, Promega) in 50 ul of Opti-MEM I before adding into the culture in 1 ml of appropriate growth media. The cells were left in transfection media overnight before proceeding with further experimentation.

For the generation of stable lines with Dsg3 knockdown, lentiviral shRNAs (containing three hits, Dharmacon) were used. Transfection of NTERT cells was carried out following the manufacturer’s instruction. Briefly, cells were seeded in 24-well plate at a density of 5x10^4^ in appropriate growth medium which was replaced, the next day, with the appropriate amount of virus suspended in serum-free medium. After 4 hours incubation, 1 ml of growth medium supplemented with serum was added. After 48 hours of post-transduction, cells were examined under a fluorescent microscope for the presence of a reporter gene (TurboGFP+ cells at approximately 30-40%). Then, all cells, including a non-target control and three hits, were treated with puromycin (at the concentration of 0.5 µg/ml) for 2 weeks and the medium containing puromycin was replaced every other day. The concentration of the drug was determined by a dose-response curve. Knockdown of Dsg3 was confirmed by Western blotting analysis that revealed only one out of three hits had a successful knockdown of Dsg3. Cells were propagated for later immunofluorescence and the treatment with UV and MG-132, respectively.

**Immunofluorescence staining and image analysis**

All immunofluorescent staining was performed in cells seeded on coverslips. Briefly, the siRNA pre-treated or untreated cells were seeded on coverslips at various densities according to the experiments and incubated in normal keratinocyte growth medium for different time periods. For p53 staining, coverslips were fixed with ice-cold methanol for 10 minutes at room temperature (RT) and then were washed in PBS twice before staining. Immunostaining was performed following our standard protocol^6^; the nonspecific binding sites were blocked for 15-30 minutes with 10% goat serum before the primary and then the secondary antibody incubation, each lasted for 1 hour at RT. Coverslips were washed 3 times with washing buffer (PBS containing 0.2% Tween 20) after each antibody incubation and were counterstained with DAPI for 8–10 minutes before the last wash and then mounted on slides. Images of fluorescent staining were acquired at the same exposure for each channel, with a 40x oil objective in Leica DM4000 Epi-Fluorescence microscope or a 63x oil objective in Zeiss 710 Laser Scanning Confocal Microscope. Super-resolution microscopy was performed using a Zeiss 880 Laser Scanning Confocal Microscope. Image analysis was performed with ImageJ. For the quantitation of p53 nuclear-positive staining, firstly, each image was subtracted with its binary image of DAPI channel before measurement of cytoplasmic signals. Then, the nuclear signals were calculated by subtracting the cytoplasmic signals from the total IMF and finally, the average per cell was determined by dividing total nuclear signals with the cell number in each field in an Excel spreadsheet before statistical analysis. Data were presented as the average IMF per cells in the final plots.

The detail procedures for immunostaining of mouse back skin were described previously^7^. Two mice in each group, i.e. Dsg3+/- and Dsg3-/-, were included in the study. Before sectioning, the back skin tissue was stored in DMEM at -80°C and embedded into Tissue Tec Freezing medium (Jung). 5 µm thick frozen sections were placed on Silane-Prep slides (Sigma-Aldrich Chemie GmbH, München, Germany). The sections were fixed with a paraformaldehyde-lysine-periodate solution and stained with antibodies directed against p-p53 (Santa Cruz, 1:50), active caspase-3 (R&D Systems, 1:50) and p21^WAF1/CIP1^ (Abcam, 1:100). For detection, secondary antibodies (anti-goat, anti-rabbit, and anti-rat) tagged to Cy3 or Alexa488 (Dianova, 1:500) were used. Nuclei were counterstained with DAPI (Sigma). Images were acquired with a Zeiss LSM 880 confocal laser microscope (40x and 63x objectives). For counting of positive hair follicles in the back skin of Dsg3^-/-^ and Dsg3^+/-^ mice, multiple images for each follicle were acquired using a 5x objective and then assembled in Adobe Photoshop CS6 (Adobe). Hair follicles with positive cells for p53/p21^WAF1/CIP1^ or p53/caspase3 were marked and scored. Statistical analysis was obtained by two-sided (two-tailed) Fisher’s exact test.

**Immunohistochemistry in PV specimens**

Oral tissue samples from 25 PV patients and 10 normal individuals, as well as 3 oral cancer patients, were analyzed by immunohistochemistry with the mouse anti-p53 (ZM-0408) antibody. Paraffin-embedded tissue sections were deparaffinized, hydrated, and heated in EDTA based antigen retrieval solution (pH=8). After washing 3 times in PBS, tissue sections were subjected to incubation in 3% H_2_O_2_ in PBS for 10 minutes at RT followed by three washes in PBS. Then slides were incubated with the primary antibody for p53 (1:100 dilution) at 4°C overnight. The next day, sections were incubated for additional 30min at 37°C before washing 3x followed by incubation with the secondary antibody (PV-6002) for 30min at 37°C. Finally, the antibody binding was detected by incubating the tissue slides in a solution of DAB before mounting. Immunohistochemical positivity was evaluated by two independent pathologists using scoring criteria. Each section was scored by counting the positive among 100 cells per field with a high-power objective; 5 arbitrary fields were selected for scoring. According to the percentage ranges of positivity, the frequencies of expression were categorized into five grades; 0: <5% positive cells; 1: 5~25% positive cells; 2: 25~50% positive cells; 3: 50~75% positive cells; 4: >75% positive cells. The staining intensity was categorized into four grades; 0: nil staining; 1: weak yellow staining; 2: staining in yellowish-brown; and 3: brown. The final score was calculated based on the above two categories and thus, scores 0-1 were defined as negative and score ≥2 were defined as positive.

**Western blotting analysis and Co-Immunoprecipitation assay (Co-IP)**

Western blotting analysis and Co-immunoprecipitation assay were conducted following procedures previously described^2,5,6,8^. Briefly, cell extraction was performed to isolate proteins from cultures at approximately 90% confluence. The culture was washed with ice-cold PBS and lysed on ice with 2x sodium dodecyl sulfate (SDS) laemmli sample buffer (0.5M Tris-Cl pH6.8, 4%SDS, 20% Glycerol; 10% (v/v) 2-mercaptoethanol was added after protein assay). DC protein assay (Bio-Rad, Hertfordshire, UK) was used to determine the protein concentration for each sample. An equal amount of proteins (10 ug) were separated by SDS-PAGE at 100- 150 V, followed by transfer to nitrocellulose membrane (10600002, GE Healthcare) at 30 V in an electrophoresis apparatus. The non-specific binding sites on the membrane were blocked for 20-30 min at RT in blocking buffer prepared as 5% (w/v) non-fat dry milk in TTBS (Tris buffer containing 0.1% Tween 20 (P1379. Sigma)) and the membranes were incubated with the primary antibodies against specific proteins at appropriate dilutions for each antibody, for overnight at 4°C. After three washes in TTBS, the membranes were incubated with the secondary antibodies conjugated with HRP (rabbit-A6667, mouse-A0168, Sigma) with 1:1000 dilution in blocking buffer for one hour at RT. After three washes in TTBS, the membranes were subjected to the chemiluminescent solution-Amersham ECL plus Western blotting detection system (28906836, GE Healthcare) following the manufacturer’s instructions. The membrane was then exposed to Amersham Hyperfilm ECL and developed in an AGFA Curix 60 developer in a dark room to detect target proteins. The band densitometry of blots were performed in ImageJ software and compared between each sample after normalization with an internal loading control. For co-IP, the same protein extraction and quantification protocol was used except that the buffer used was 1x RIPA buffer (Upstate) containing a protease inhibitor cocktail (Calbiochem). For immunoprecipitation, 1mg of protein was incubated with 5 µg of primary antibody at 4°C overnight on a rotation wheel. The following day, 1.7x10^5^ Protein G magnetic Dynabeads® (10003D, Invitrogen) was added to the Lysate: antibody solution and incubated for a further 1 hour at 4° C on a rotation wheel. The bead-bound immune precipitates were washed 4x in RIPA buffer and 1x in 1xTTBS. The resulting immune precipitates were re-suspended in 11 µl of 2x sample buffer and boiled for 2 min at 95-100°C and finally, were resolved and analyzed in the same manner as described for Western blotting.

**Nuclear Extraction**

Nuclear protein extraction was carried out to separate the nuclear and cytoplasmic fractions. Pre-chilled reagents and glassware were used to minimize protein degradation. Cells were collected in PBS by scrapping the cells off the surface of the plate with a cell scraper. Harvested cells were centrifuged at 600 xg for 5 mins, followed by washing with PBS twice and then the supernatant was carefully removed and discarded leaving behind the cell pallet. Cells were gently re-suspended in the hypotonic buffer by pipetting up and down several times, which bursts the cell wall open, while keeping the nuclear membrane intact. This was followed by adding detergent (10% NP40) and then vortexing to separate the nuclei from the cytoplasmic fraction. Homogenate was then centrifuged to yield the cytoplasmic fraction in the supernatant and the remaining pallet was the nuclear fraction. The nuclear pallet was re-suspended in the cell extraction buffer for 30 mins on ice with vortexing at 10 mins intervals. This bursts the nuclear membrane. This was finally centrifuged at high speed for 30 mins to yield the nuclear fraction in the supernatant. Extracted proteins were quantified by DC protein assay and analyzed by Western blotting as described above.

**FACS based Cell Viability-Caspase-3 assay**

This assay was followed by the procedures described previously^9^. Briefly, harvested cells were labeled with fixable live dead stain, Zombie NIR (Near Infra-Red) (BioLegend, UK) for 15 min, then washed in PBS/BSA and fixed in Solution A (Cal-Tag, UK) for 15 min. Washed cells were permeabilized in 0.25% Triton X-100 (Sigma, UK) for 15 min followed by incubation with anti-active caspase-3-BV650 (Cat. No. 564096, BD Biosciences, USA) for 20 min. All procedures were performed at RT. Cells were re-suspended in PBS-400 and analyzed on an ACEA Bioscience Novocyte 3000 flow cytometer. Cells were gated on a dot-plot of Caspase-3-BV650 vs Zombie NIR with a quadrant placed marking off live cells in the double negative quadrant (lower left), with Caspase-3-BV650^+ve^/Zombie NIR^-ve^ (lower right) and lastly with Caspase-3-BV650^+ve^/Zombie NIR^+ve^ and Caspase-3-BV650^-ve^/Zombie NIR^+ve^ upper quadrants indicating dead cells.

**Luciferase assay**

Cells with either Dsg3 knockdown or ectopic overexpression, seeded in 24-wells were transfected with p53 luciferase reporter (Plasmid #28175: 145-pGL3ctrl-3' UTR, Addgene) using Fugene 6 transfection reagent at 0.25 ug per well. After 24h incubation, lysates were harvested prior to the luciferase assay using the Luciferase Assay System (Promega)^8^. Finally, luciferase activities were normalized against protein concentration determined by Bio-Rad DC protein assay (Bio-RadLaboratories Ltd., Hertfordshire, UK).

**RT-qPCR**

The detail procedure in reverse transcription quantitative PCR (RT-qPCR) analysis was described previously^10^. Briefly, the mRNA harvested using Dynabeads mRNA Direct kit (Invitrogen) was converted to cDNA using qPCRBIO cDNA Synthesis kit (#PB30.11-10, PCRBIO Systems, UK) and the cDNA was diluted 1:5 with RNase/DNase free water and stored at -20°C until used for qPCR. Relative gene expression qPCR was performed using qPCRBIO SyGreen Blue Mix Lo Rox (#PB20.11-50, PCRBIO Systems, UK) in the 384-well LightCycler 480 qPCR system (Roche) according to our well-established protocols^10^ which are MIQE compliant. Thermocycling begins with 95ºC for the 30s prior to 45 cycles of amplification at 95ºC for 1s, 60ºC for 1s, 72ºC for 6s, 76ºC for 1s (data acquisition). A ‘touch-down’ annealing temperature intervention (66ºC starting temperature with a step-wise reduction of 0.6ºC/cycle; 8 cycles) was introduced prior to the amplification step to maximize primer specificity. Melting analysis (95ºC for the 30s, 65ºC for 30s, 65-99ºC at a ramp rate of 0.11ºC/s) was performed at the end of qPCR amplification to validate single product amplification in each well. Relative quantification of mRNA transcripts was calculated based on an objective method using the second derivative maximum algorithm (Roche). All target genes were normalized using a stable reference gene (POLR2A). Samples were presented as the mean ± SEM of 3 replicates. The primers used in the study are shown in Table 1.

**Supplementary Table**

Table S1. Primer sequences of keratin and terminal differentiation genes analyzed in the study

|  | **Gene** | **Product size (bp)** | **Forward primer** | **Reverse primer** |
| --- | --- | --- | --- | --- |
| **Control** | POLR2A | 73 | GCAAATTCACCAAGAGAGACG | CACGTCGACAGGAACATCAG |
| **Target genes** | DSG3 | 164 | CCGAATCTCTGGAGTGGGAA | GCCCAAGGACTAGATGTAGA |
|  | TP53 | 85 | aggccttggaactcaaggat | ccctttttggacttcaggtg |
|  | K1 | 128 | CGGAACTGAAGAACATGCAG | CATATAAGCACCATCCACATCC |
|  | K4 | 134 | TCCTGAAGGTCCTCTATGATGC | GTACTGGGCACGGACCTC |
|  | K8 | 119 | GATGAACCGGAACATCAGC | CATCCTTAATGGCCAGCTCT |
|  | K10 | 134 | AAACCATCGATGACCTTAAAAATC | GCGCAGAGCTACCTCATTCT |
|  | K13 | 100 | AGTCCCAGCTGAGCATGAA | CTGCTGATGAGTCCCTGGAT |
|  | K14 | 124 | CGACCTGGAAGTGAAGATCC | GTCCACTGTGGCTGTGAGAA |
|  | K18 | 112 | TGATGACACCAATATCACACGA | GGCTTGTAGGCCTTTTACTTCC |
|  | K19 | 126 | GCCACTACTACACGACCATCC | CAAACTTGGTTCGGAAGTCAT |
|  | K20 | 108 | CCTCAAAAAGGAGCATCAGG | ATGATGACGCCAAGGTTCA |
|  | K23 | 113 | GCAGACACAGTACAGCACGAA | CCTCCTCATAGTGGGAGATGA |
|  | CORN | 90 | tcactgttgcagcatgagttc | tggcaaggctgtttcacc |
|  | IVL | 83 | tgcctgagcaagaatgtgag | ttcctcatgctgttcccagt |
|  | FLG | 92 | CAAGTCCAGGAGAGACACG | TGCAGATGAAGCTTGTCCAC |

**Supplementary figures**

Fig. S1 Keratinocyte differentiation marker showed an inverse relationship with Dsg3 expression levels. Four different cell lines with either Dsg3 knockdown or overexpression were assessed by RT-qPCR and the results signify an inverse relationship between Dsg3 expression levels and various cell differentiation markers. Heat map of Log2 fold changes of knockdown (KD) or overexpression against to the respective controls, for the indicated genes, i.e. keratin and terminal differentiating structural genes, in various cell lines, such as NTERTs with Dsg3 knockdown, and three lines (cutaneous keratinocyte line T8, oral keratinocyte line SqCC/Y1 and colorectal HCT116 cells) with exogenous Dsg3 overexpression; n=3 biologically independent samples, asterisks indicate statistical significance via unpaired two-sided student t-test, with p <0.05.

Fig. S2. Overexpression of Dsg3 in various cell lines protects cells from the UV induced cell death. **a** Analysis of p53 and its targets p21^WAF1/CIP1^ and Bax in stable MDCK cell lines (harbor wtp53), Vect control and D3 with overexpression of Dsg3, without or with UV irradiation (n=3 biologically independent samples). Phase contrast images below showed the loss of a large subpopulation of Vect cells one day after UV whereas, in contrast, many D3 cells remained attached to the substrate. Scale bar, 50 µm. **b** A2780 (ovary cancer line) and HCT116 (colorectal cancer line) that harbor wtp53 were seeded in 6 -well plates for 1 day before treated in the absence or presence of UVB irradiation (10mj/cm^2^). After 1 day, some UV treated cells were floating. All the attached cells in quadruplicate wells (V and D3, -/+ UV) in each condition were harvested by trypsin/EDTA and the total cell number in each well was determined by direct cell counting with a CASY machine (n=3, data are mean±s.d.). Significantly increased number of viable cells were detected in the Dsg3 overexpressing cell lines compared to the respective controls. All comparisons were made using unpaired two-sided student t-test, *p<0.05, ***p<0.001.

Fig. S3 p53 knockdown results in a significant reduction of p53 staining signals in cells treated with PV sera. Cells seeded in sparse densities were transfected with either scrambled siRNA or p53 siRNA before being harvested and re-plated on coverslips at sub-confluent densities for overnight. Then, cells were treated with PV sera or control sera (40% in keratinocyte growth medium) for 24 hours prior to fixation and immunostaining for p53. In control of serum treated samples, the p53 staining was almost completely abrogated by p53 knockdown. In contrast, some high background was detected in PV serum-treated cells with p53 knockdown (Serum8). However, statistically significant differences were still shown in both the total and nuclear p53 between control serum-treated cells and PV serum treated samples, as well as between control siRNA treated and p53 knockdown cells exposed to each PV patient serum (n=5~14, data are mean±s.d., *p<0.05, **p<0.01, ***p<0.001). Scale, 10µm.

Fig. S4 Both Dsg3 knockdown and PV sera treatment cause increased Bax expression in cells, in the cytoplasm and/or nucleus. Confocal microscopy of Bax and Dsg3 (rabbit Ab H145) staining in NTERT cells treated with siRNA (**a**) and T8-D3 cells treated with PV sera (**b**). In the latter, cells were transfected with a wtp53 plasmid for 1 day before being harvested and seeded on coverslips and then treated with PV sera (40% in KGM) for 24 hours. The quantitation data for both Bax (including cytoplasmic, as indicated by arrows, and nuclear staining signals) and Dsg3 are shown in the bar charts on the right. (n>4, mean±s.d., *p<0.05, ***p<0.001). Scale, 10µm.

**References**

1. Mannan, T. et al. RNAi-mediated inhibition of the desmosomal cadherin (desmoglein 3) impairs epithelial cell proliferation. *Cell Prolif.* **44**, 301-310 (2011).

2. Tsang, S. M. et al. Non-junctional human desmoglein 3 acts as an upstream regulator of Src in E-cadherin adhesion, a pathway possibly involved in the pathogenesis of pemphigus vulgaris. *J Pathol.* **227**, 81-93 (2012).

3. Tsang, S. M. et al. Desmoglein 3 acting as an upstream regulator of Rho GTPases, Rac-1/Cdc42 in the regulation of actin organisation and dynamics. *Exp. Cell Res.* **318**, 2269-2283 (2012).

4. Wan, H., A. P. South, & I. R. Hart. Increased keratinocyte proliferation initiated through downregulation of desmoplakin by RNA interference. *Exp. Cell Res.* **313**, 2336-2344 (2007).

5. Tsang, S. M. et al. Desmoglein 3, via an interaction with E-cadherin, is associated with activation of Src. *PLoS. One.* **5**, e14211 (2010).

6. Moftah, H. et al. Desmoglein 3 regulates membrane trafficking of cadherins, an implication in cell-cell adhesion. *Cell Adh Migr.* 1-22 (2016).

7. Hunefeld, C. et al. Bone Marrow-Derived Stem Cells Migrate into Intraepidermal Skin Defects of a Desmoglein-3 Knockout Mouse Model but Preserve their Mesodermal Differentiation. *J. Invest Dermatol.* **138**, 1157-1165 (2018).

8. Brown, L. et al. Desmoglein 3 promotes cancer cell migration and invasion by regulating activator protein 1 and protein kinase C-dependent-Ezrin activation. *Oncogene* **33**, 2363-2374 (2014).

9. Lee, H. L. et al. Simultaneous flow cytometric immunophenotyping of necroptosis, apoptosis and RIP1-dependent apoptosis. *Methods* **134-135**, 56-66 (2018).

10. Gemenetzidis, E. et al. FOXM1 upregulation is an early event in human squamous cell carcinoma and it is enhanced by nicotine during malignant transformation. *PLoS. One.* **4**, e4849 (2009).
