# Supplementary figures and images for: The desmosomal cadherin desmoglein-3 acts as a keratinocyte anti-stress protein via suppression of p53
Source: Cell Death Dis. 2019 Oct 3;10(10):750. doi: 10.1038/s41419-019-1988-0 (PMC6776551; doi:10.1038/s41419-019-1988-0)

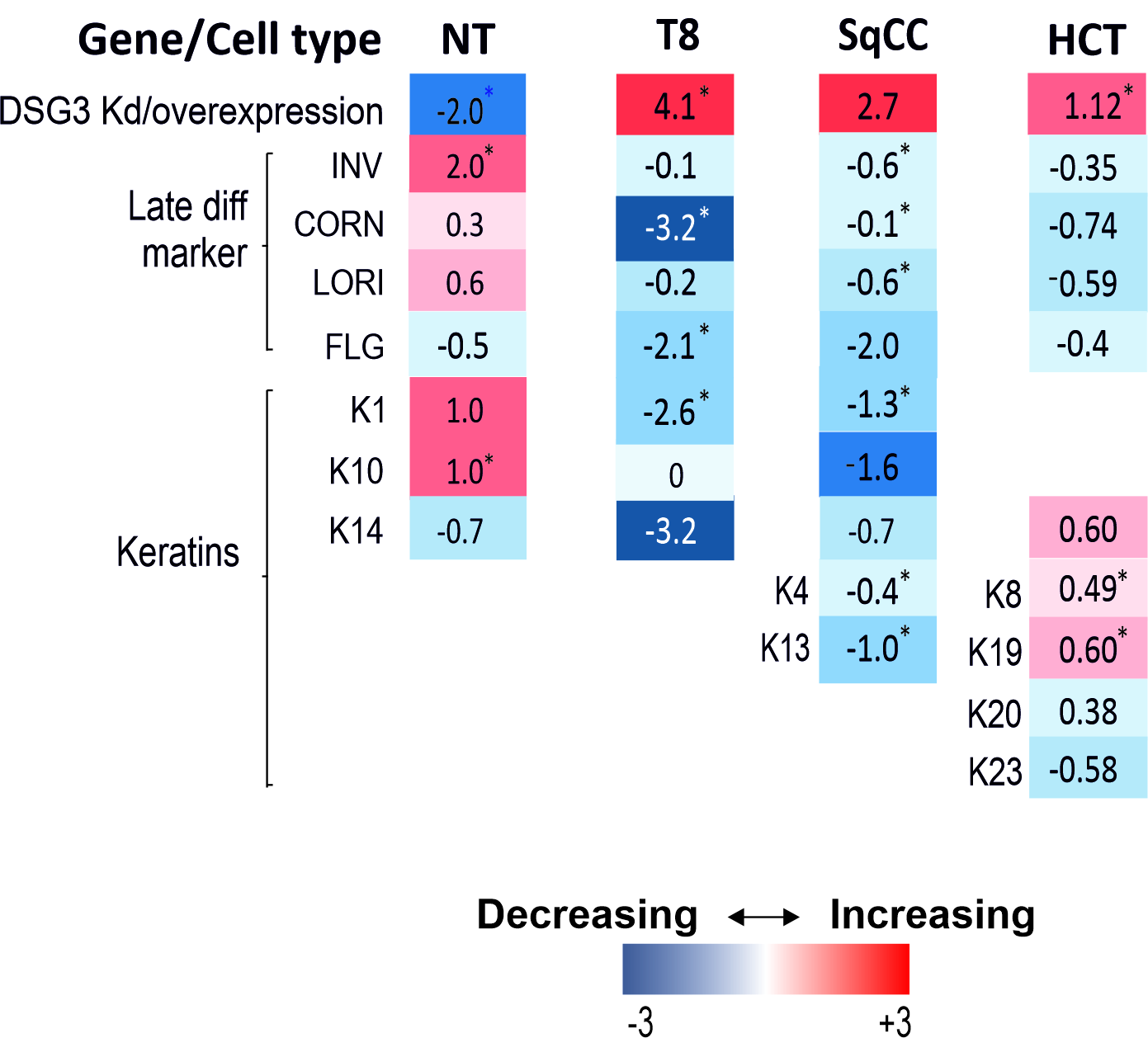

Supplement: Supplementary file 2 — Fig. S1. Keratinocyte differentiation marker showed an inverse relationship with Dsg3 expression levels [file 41419_2019_1988_MOESM2_ESM.tif]

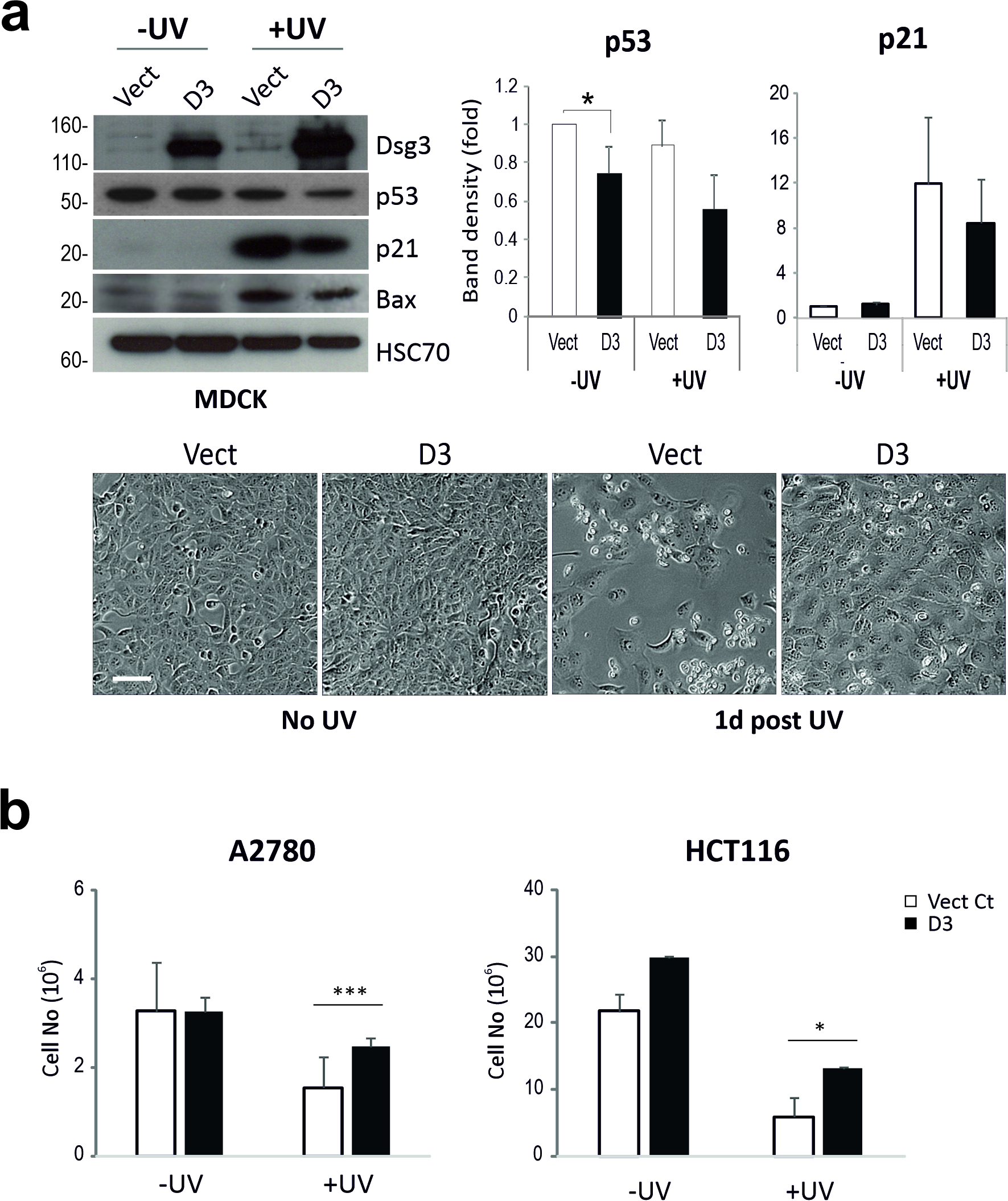

Supplement: Supplementary file 3 — Fig. S2. Overexpression of Dsg3 in various cell lines protects cells from the UV induced cell death [file 41419_2019_1988_MOESM3_ESM.tif]

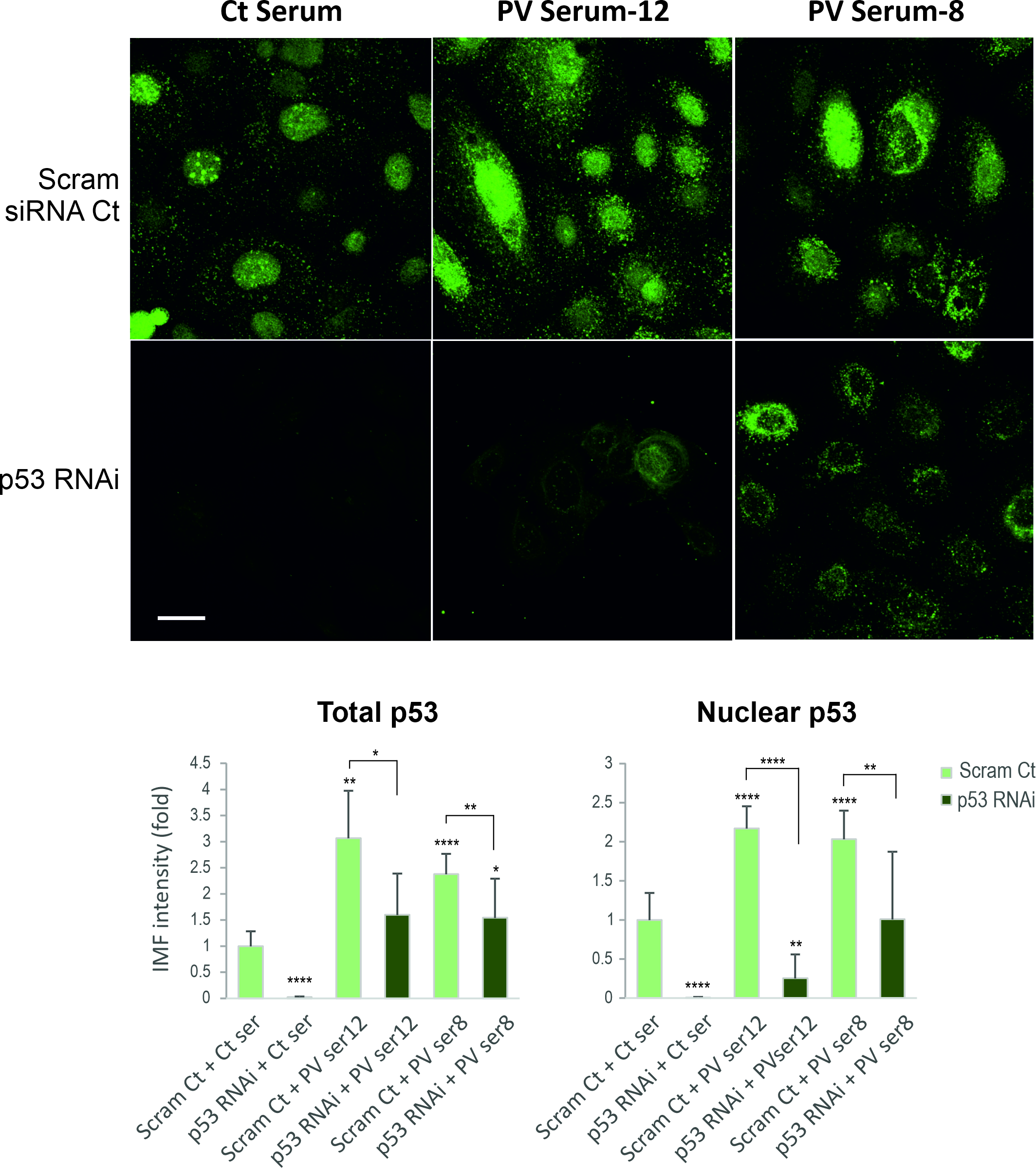

Supplement: Supplementary file 4 — Fig. S3. p53 knockdown results in a significant reduction of p53 staining signals in cells treated with PV sera [file 41419_2019_1988_MOESM4_ESM.tif]

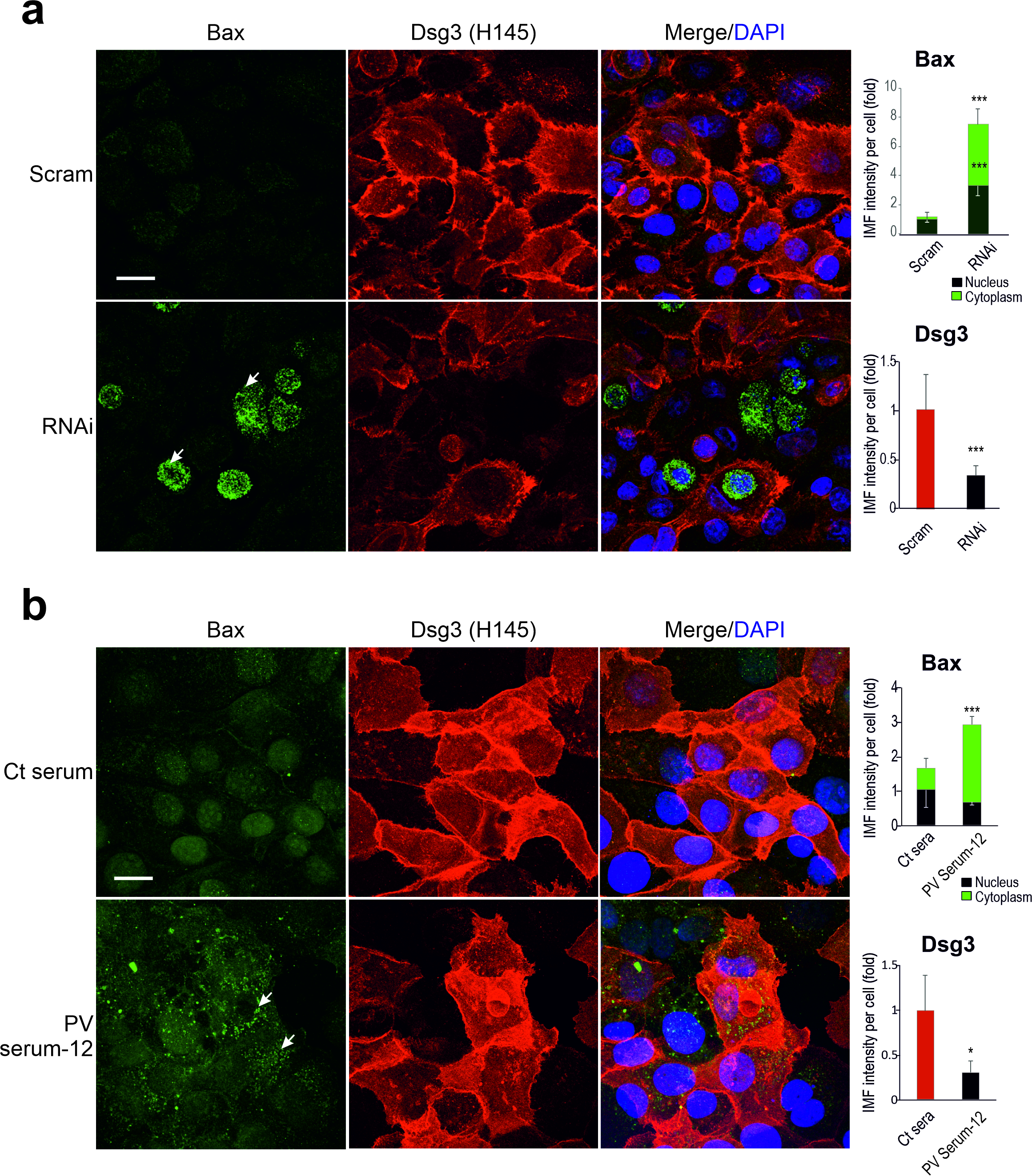

Supplement: Supplementary file 5 — Fig. S4. Both Dsg3 knockdown and PV sera treatment cause increased Bax expression in cells, in the cytoplasm and/or nucleus [file 41419_2019_1988_MOESM5_ESM.tif]
